# Signaling Pathways and Sex Differential Processes in Autism Spectrum Disorder

**DOI:** 10.3389/fpsyt.2021.716673

**Published:** 2021-10-08

**Authors:** Kristen D. Enriquez, Abha R. Gupta, Ellen J. Hoffman

**Affiliations:** ^1^Program on Neurogenetics, Child Study Center, Yale University School of Medicine, New Haven, CT, United States; ^2^Department of Pediatrics, Yale University School of Medicine, New Haven, CT, United States; ^3^Department of Neuroscience, Yale University School of Medicine, New Haven, CT, United States

**Keywords:** autism spectrum disorder, female protective effect, estrogens, imaging genomics, animal models, genetics

## Abstract

Autism spectrum disorders (ASDs) are a group of neurodevelopmental disorders associated with deficits in social communication and restrictive, repetitive patterns of behavior, that affect up to 1 in 54 children. ASDs clearly demonstrate a male bias, occurring ~4 times more frequently in males than females, though the basis for this male predominance is not well-understood. In recent years, ASD risk gene discovery has accelerated, with many whole-exome sequencing studies identifying genes that converge on common pathways, such as neuronal communication and regulation of gene expression. ASD genetics studies have suggested that there may be a “female protective effect,” such that females may have a higher threshold for ASD risk, yet its etiology is not well-understood. Here, we review common biological pathways implicated by ASD genetics studies as well as recent analyses of sex differential processes in ASD using imaging genomics, transcriptomics, and animal models. Additionally, we discuss recent investigations of ASD risk genes that have suggested a potential role for estrogens as modulators of biological pathways in ASD, and highlight relevant molecular and cellular pathways downstream of estrogen signaling as potential avenues for further investigation.

## Introduction

Autism spectrum disorders (ASDs) are a group of neurodevelopmental disorders characterized by persistent deficits in social interaction and communication and by the presence of restricted, repetitive patterns of behavior, interests, or activities (1). While the clinical presentation of ASDs may be highly heterogeneous, individuals with ASD often experience difficulties with social-emotional reciprocity and non-verbal communication, stereotyped movements, rigid adherence to routines, and hyper- or hypo-sensitivity to sensory stimuli (1). At present, there are no approved pharmacological treatments that target these core deficits, which is due in part to our limited understanding of their underlying pathophysiology. ASDs have been increasing in prevalence in recent years, with estimates indicating that 1 in 54 children in the United States is affected (2), underscoring the need for improved insights into ASD biology. Recent human gene discovery efforts have been instrumental in this regard, with large-scale whole-exome sequencing studies leading to the identification of at least 102 genes that are strongly associated with risk (3–6). Interestingly, while ASD risk genes encode proteins involved in seemingly divergent functions, such as ion channels, synaptic cell adhesion molecules, and chromatin modifiers, there is growing evidence that these genes converge on common neurodevelopmental pathways, such as the regulation of gene expression and neuronal communication (6), which are likely to play a central role in ASD biology.

Despite this progress in gene discovery, gaps in our understanding of the biology of ASD remain. ASDs show a clear male bias, occurring at least four times more often in males than females, yet the basis for this male predominance is not well-understood (2). While ascertainment bias favoring the recognition and diagnosis of ASD in males, as well as “camouflaging” of social deficits by females, might contribute, ASD is likely more common in males even after accounting for these factors (7). Interestingly, there is growing evidence from human genetics analyses supporting a “*female protective effect*” in ASD. The female protective effect theory assumes that the risk for ASD is quantitative and follows a normal distribution in the general population, but that females are protected against this risk and thus have a higher liability threshold for ASD diagnosis (5, 7). While the etiology of the female protective effect in ASD is unknown, one hypothesis is that differential exposure to sex steroid hormones may be a contributing factor (7). Interestingly, recent studies analyzing the function of ASD risk genes in animal models, including one from our group, independently identified estrogens as suppressors of ASD gene-associated cellular and behavioral phenotypes, implicating estrogens as potential modulators of biological pathways relevant to ASD (8, 9). However, studies directly assessing the role of estrogens in ASDs, both in clinical and preclinical models, are limited.

Here, we provide an overview of signaling pathways and mechanisms implicated by ASD genetics studies, as well as sex differential biological processes that may contribute to the female protective effect. In addition, we review clinical and preclinical studies suggesting a role for estrogens in ASD and discuss potential mechanisms by which estrogens might interact with relevant signaling pathways and neural cell types. While the effect of environmental exposure to estrogens on brain development has been an area of interest, this is beyond the scope of this review and has been reviewed elsewhere (10, 11). In this review, we focus on signaling pathways that have been implicated by studies of ASD risk genes and consider potential interactions between these pathways and established mechanisms of endogenous estrogen signaling in the brain. Taken together, investigations into the etiology of the increased male to female ratio in ASD—a central feature of ASD—are likely to inform our understanding of ASD pathophysiology more broadly and may provide novel avenues for pharmacological intervention.

## ASD Risk Gene Discovery

ASD risk gene discovery has been accelerating at a rapid pace in recent years, made possible by advances in genomic sequencing technologies. Large-scale, whole-exome and whole-genome sequencing studies have led to the identification of a growing list of “high confidence” genes that are strongly associated with ASD and are beginning to reveal common biological pathways. ASDs are known to be highly heritable, with greater concordance rates among monozygotic (50–90%) compared to dizygotic (up to 30%) twins (12–18). Early efforts at gene discovery in ASD led to the identification of genes associated with rare monogenic disorders, including Fragile X syndrome (*FMR1*), tuberous sclerosis complex (*TSC1, TSC2*), Rett syndrome (*MECP2*), and *PTEN* hamartoma tumor syndrome (*PTEN*) [reviewed in (19)]. Additional early studies using molecular cytogenetics and sequencing approaches led to the discovery of some of the first ASD-associated genes, including *NLGN4X* and *NRXN1*, which encode synaptic cell adhesion molecules (20–24), and *SHANK3*, encoding a synaptic scaffolding protein (25, 26).

While common variants likely account for the majority of the inherited risk of ASD (27–29), most progress in gene discovery in recent years, and all of the “high confidence” risk genes, were discovered through the identification of rare variants of large effect (occurring in <0.1% of the general population) (3–6). These studies involved whole-exome sequencing of individuals from multiple consortia, including the Autism Sequencing Consortium, the iPSYCH-Broad Consortium, the Simons Simplex Collection (SSC), and others. In particular, the Simons Simplex Collection is a cohort of simplex families consisting of an affected child, unaffected parents, and in most cases, an unaffected sibling, thus increasing the likelihood of identifying rare, *de novo* (not inherited) variants. Importantly, these studies found that rare *de novo*, likely gene-disrupting single-nucleotide variants (SNVs) occur more frequently in individuals with ASD than in unaffected siblings, and that the genes affected by such variants when recurrent in unrelated individuals represent bona fide risk genes (30).

This finding was consistent with the earlier discovery that *de novo* copy number variants (CNVs), i.e., microdeletions and microduplications, also occur significantly more often in individuals with ASD, particularly from simplex families, than unaffected individuals (31). The increased rate of rare and *de novo* CNVs in ASD was confirmed in subsequent investigations (5, 32, 33), which led to the discovery of additional ASD-associated genes and genomic regions, such as 15q11.2-13, 16p11.2, and 22q11.2 (5, 22, 24, 32–36). It is estimated that *de novo* CNVs and SNVs collectively account for ~30% of simplex cases (5). Together, these groundbreaking studies led to the identification of the first “high confidence” ASD risk genes and shaped our understanding of the genetic architecture of ASDs, establishing a clear contribution of rare, *de novo* variants to ASD risk and a path forward for gene discovery (3–6, 30, 37, 38). SFARI Gene (https://gene.sfari.org/), an excellent resource established by the Simons Foundation for Autism Research Initiative (SFARI), is a database that ranks ASD risk genes based the strength of the evidence supporting their association with ASD on a 1–3 scale, where genes with score 1 have the strongest evidence for association (39).

## Biological Pathways Implicated by ASD Risk Genes

With expanding DNA-sequencing efforts, the number of ASD-associated genes has continued to climb, enabling the identification of related biological pathways involving these genes. In the largest exome-sequencing study of ASD to date, involving almost 12,000 individuals from family-based and case-control samples, Satterstrom et al. (6) identified 102 ASD risk genes (false discovery rate < 0.1) and examined biological pathways involving these genes. Specifically, they found that the 102 risk genes are expressed early in the developing brain and fall broadly into the following functional categories: gene expression regulation, neuronal communication, cytoskeleton, and other functions (6). “Gene expression regulation” includes chromatin modifiers and transcription factors, while “neuronal communication” encompasses a range of functions, including ion channels, synaptic cell adhesion molecules, intracellular signaling molecules, and axon guidance molecules (6). These findings are consistent with earlier studies implicating synaptic function as a central mechanism in ASD, based on the identification of risk genes encoding synaptic cell adhesion molecules and scaffolding proteins, including neurexins, neuroligins, and SHANK proteins (20–26). Additionally, the first ASD risk genes associated with monogenic disorders, e.g., *TSC1, TSC2, FMR1, NF1*, and *PTEN*, are directly or indirectly involved in mechanistic target of rapamycin (mTOR) signaling, a central pathway controlling protein translation, cellular proliferation, and survival, suggesting that mTOR may represent another common pathway in ASD (19).

Recent studies have also shown that ASD risk genes encode proteins involved in transcriptional regulation, chromatin remodeling, and synaptic formation (3, 4). A protein-protein interaction network of high confidence ASD risk genes and predicted risk genes also identified cell-cell communication, synaptic transmission, and transcriptional regulation as relevant clusters (3). Interestingly, Iossifov et al. (4) observed that the targets of *de novo*, likely gene disrupting mutations overlapped significantly in females and males with a lower intelligence quotient (IQ), but not males with a higher IQ. They found that this overlapping group of genes is enriched for embryonically expressed genes (mainly in females), suggesting an early developmental effect, as well as chromatin modifiers and targets of FMRP (encoded by *FMR1*), the Fragile X syndrome RNA-binding protein. Likewise, Pinto et al. (32) found that females with ASD are more likely than males to carry highly penetrant CNVs and CNVs disrupting FMRP targets. This suggests that females are more likely to carry mutations of greater impact and that less deleterious mutations show decreased penetrance in females, consistent with the female protective effect (discussed in the next section) (4, 7, 32).

Integrative genomics approaches aimed at analyzing expression patterns of ASD risk genes have also played a critical role in identifying relevant neural cell types and developmental time points. For example, Willsey et al. (40) utilized human brain transcriptome data from the BrainSpan project (41) encompassing early embryonic to late adult stages and multiple brain regions to construct spatio-temporal co-expression networks around nine high confidence ASD genes, and identified mid-fetal glutamatergic deep layer projection neurons as a point of convergence (40). Another study analyzed the same transcriptome dataset using weighted gene co-expression network analysis (WGCNA) (42), and remarkably, also found that ASD risk genes are enriched in glutamatergic projection neurons, though in superficial, not deep, layers (43). Further, an imbalance in excitatory-inhibitory signaling in the brain has previously been proposed as a central mechanism underlying ASD (44). By highlighting excitatory glutamatergic neurons as a point of convergence, these studies provide further support for this theory. The recent exome-sequencing study by Satterstrom et al. (6) also provides evidence for excitatory-inhibitory imbalance; using a single-cell RNA sequencing dataset from prenatal human forebrain (45), this study found that ASD risk genes are enriched in maturing and mature excitatory and inhibitory neurons, with the strongest enrichment in early excitatory neurons and striatal interneurons (6). Taken together, these studies highlight neuronal communication, synaptic transmission, and regulation of gene expression as convergent biological processes involving ASD risk genes and implicate excitatory and inhibitory neurons as neural cell types with relevance to ASD pathophysiology.

## The Female Protective Effect in ASD

As discussed above, large-scale genetic studies have been productive in identifying genes strongly associated with ASD. They have also yielded the intriguing observation that females with ASD carry, on average, more deleterious mutations compared to males with ASD (46). That is, females with ASD are more likely to have an increased mutation “burden.” For example, Sanders et al. (5) found that *de novo* CNVs occur at a greater rate in females with ASD. In addition, Satterstrom et al. (6) observed that *de novo*, protein-truncating variants in the most strongly associated ASD risk genes show a two-fold enrichment in females compared to males, consistent with findings from earlier exome-sequencing studies (3, 4). The apparent implication that females require a larger etiological “hit” to develop ASD supports the “female protective effect” model, which posits that there are innate factors that cause females to be relatively resilient to the disorder, leading to the overrepresentation of males in ASD. Still, the sex differential in diagnosis could be due to factors that cause males to be especially vulnerable to developing ASD as well as factors that cause females to be resilient to developing the condition; they are not mutually exclusive.

While there is highly suggestive evidence from genetic analyses supporting a female protective effect in ASD, the biological mechanisms responsible for female protection and/or male risk remain unclear. It has been proposed that multiple “hits,” involving some combination of sex, genetics, and environmental triggers, lead to the imbalance of males and females in ASD (47). Interestingly, differences in sex steroid hormone levels have been proposed as a possible mechanism contributing to resilience in females and/or increased risk in males [reviewed in (7)]. One of the most notable examples is the “extreme male brain” theory (discussed below). In addition, studies investigating the function of ASD risk genes in preclinical systems, including one from our group, identified estrogens as potential modulators of ASD gene-associated cellular and behavioral phenotypes (8, 9, 48). However, genes that directly affect sex steroid hormone signaling, e.g., estrogen or androgen receptors, have not been identified among the strongest ASD-associated genes (e.g., *ESR1*, which encodes Estrogen Receptor 1, is not listed in SFARI Gene; *AR*, encoding Androgen Receptor, has a SFARI gene score of 3), suggesting that estrogens and/or androgens may be acting indirectly to modulate signaling pathway deficits resulting from ASD risk gene mutations. Below we review investigations that have provided insights into sex differential processes and potential female protective factors in ASDs, including: genetic and transcriptomic analyses examining the sex chromosomes and sex-differential gene expression; imaging genomics studies integrating functional imaging with genetic findings; as well as clinical and preclinical studies investigating potential contributions of sex steroid hormones, with a particular focus on estrogens. Further elucidating these mechanisms will be important for identifying potential targets for treatment.

## Sex Differential Processes in ASD

### Genetic Studies

One potential explanation for the female protective effect is that it may be driven by genetic factors, i.e., specific genes that confer risk and/or protection in males or females. Although it is reasonable to presume that the male bias could be due to ASD as an X-linked disorder, chromosome X gene mutations do not account for a substantial proportion of cases and most ASD-associated genes identified to date are autosomal [(6); reviewed in (7)]. Approximately 92% of all genes in SFARI Gene are autosomal, including ~90% of those with the strongest evidence for association with ASD (SFARI gene score 1) (https://gene.sfari.org) (39). Moreover, large-scale genetic studies provide evidence that the same genes and genetic mechanisms likely contribute to ASD risk in males and females, suggesting that female protection and/or male risk may be driven by non-genetic factors. Specifically, by analyzing 10,220 individuals from 2,591 families from the SSC, Sanders et al. (5) found that *de novo*, damaging variants in high confidence ASD risk genes are randomly distributed in males and females, suggesting that a common set of genes contribute to risk in both sexes [(5); reviewed in (7)]. In addition, in the largest exome sequencing study of ASD to date with 35,584 individuals, including 11,986 with ASD, Satterstrom et al. (6) found that there is no significant difference in the types of genetic variants (i.e., protein-truncating, missense) that contribute to ASD risk in males vs. females, even though females are more likely to harbor damaging mutations in the strongest risk genes. However, there is evidence that one gene, *DDX3X*, located on the X chromosome, shows locus-specific enrichment in females (49). Interestingly, another study by Gockley et al. (50) investigated whether there is a single genetic locus that might confer resilience in females. By comparing single nucleotide polymorphisms (SNPs) on the X chromosome and genome-wide in females with and without ASD, they did not find evidence supporting a single protective genetic locus in females, though it is possible that several genetic loci may be involved (50). Taken together, large genetic studies provide strong support for the female protective model, though to date there is no clear evidence that a single gene or genetic mechanism in females or males accounts for resilience and/or risk.

### Transcriptomic Studies

To investigate how differences in gene expression might contribute to the female protective effect in ASD, Werling et al. (51) performed the first integrative genomics analysis of sex-differential gene expression with relevance to ASD. Specifically, they compared sex-differential gene expression patterns in post-mortem adult and prenatal cortex from neurotypical individuals from the BrainSpan project (adult dataset: 5 subjects of each sex, 29 samples/subject; prenatal dataset: 4 subjects of each sex, 43 samples/subject) (41) with gene expression modules from a post-mortem cortical dataset from individuals with ASD [4/16 female ASD cases, 1/16 female controls in (52); 8/32 female ASD cases and 9/41 female controls in (53)]. This study led to several key findings. First, there was no evidence for enrichment of known ASD risk genes among genes showing sex-differential expression patterns in either adult or prenatal cortex, suggesting that risk genes themselves do not show sex-differential expression (51). Second, the authors found that genes that are upregulated in the post-mortem cortex of individuals with ASD (ASD-upregulated) were significantly enriched for male differentially expressed genes, i.e., genes showing higher expression in neurotypical males. Intriguingly, the ASD-upregulated gene module is associated with astrocyte and microglial markers, indicators of immune system function, and astrocyte and microglial markers were also found to be enriched among male differentially expressed genes. In contrast, ASD-downregulated gene modules were more highly enriched for female differentially expressed genes and overlapped with synaptic and neuronal markers. Of note, this association was not likely to be due to the male predominance of the cases and controls in the post-mortem studies, given that they were sex balanced, and the potential confounds of sex were accounted for in the analyses of ASD-associated gene expression (51–53). Third, these findings were recapitulated in a prenatal neocortical dataset, including the lack of enrichment of ASD risk genes among sex-differentially expressed genes and the overlap of male differentially expressed genes and ASD-upregulated genes. This study indicates that genes whose expression is dysregulated in post-mortem brains of individuals with ASD, but not ASD risk genes themselves, show sex-differential expression patterns (51). This implies that it is the pathways downstream of ASD risk genes that might interact with sexually dimorphic biological processes to influence the sex bias in ASD (51). Furthermore, the enrichment of astrocyte and microglial markers in the male-differentially expressed and ASD-upregulated gene modules suggests that typical sex differences in immune system function may be relevant to ASD biology and will be important to investigate as a potential contributor to the sex bias in ASD in future studies (7, 51).

### Imaging Genomics

**Figure 1:**
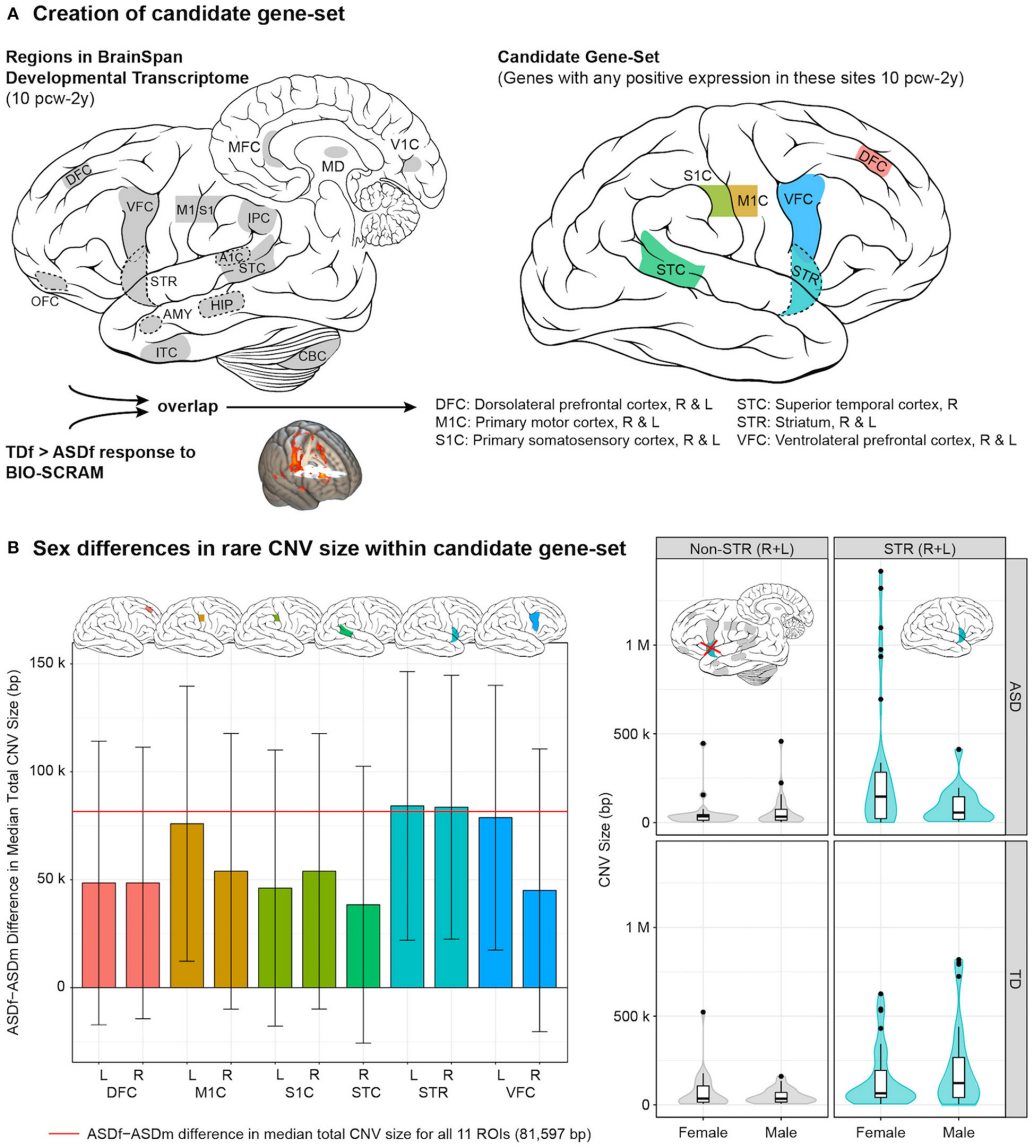
Female autism neurogenetic profile. **(A)** Creation of candidate gene-set involved identifying 11 regions of interest (ROIs) that had both been characterized in the BrainSpan Developmental Transcriptome (left) and showed a significant typically-developing female > ASD female (TDf > ASDf) response to biological motion (BIO-SCRAM); genes were then identified that showed positive expression in these overlapping regions (right) between 10 post-conceptual weeks (pcw) through 2 years (y). **(B)** (Left) bar plot with standard errors demonstrating the sex difference (ASDf - ASDm) in median total CNV size, in base pairs (bp), within the candidate gene-set. Plots of the CNV size difference are provided for each of the 11 ROIs. (Right) distribution of size of rare CNVs containing gene(s) expressed in right (R) and left (L) striatum (STR) from 10 pcw to 2 y (right panels, cyan) by group and sex. For comparison, “non-STR (R + L)” includes rare CNVs containing gene(s) characterized in BrainSpan that were not positively expressed in R/L-STR from 10 pcw to 2 y (left panels, gray). Of note, rare CNVs often contained gene(s) that were expressed in multiple brain regions (e.g., STR plus additional ROI[s]). Violin plots depict a Gaussian kernel density estimate, and are overlaid with Tukey-style boxplots. Brain art adapted from illustrations created by Patrick Lynch and C. Carl Jaffe, commons.wikipedia.org/wiki/File:Brain_human_lateral_view.svg and Brain_human_sagittal_section.svg, licensed under a Creative Commons Attribution 2.5 Generic License, 2006. Figure constructed by Allison Jack PhD and reprinted with permission from Jack et al. (54).

Imaging genomics is a cutting-edge approach to integrate neuroimaging and genomic data from individuals with ASD to gain a greater understanding of interactions between neural circuit and genetic mechanisms in ASD. Our group conducted an imaging-genomics analysis to characterize the female neural profile in ASD, which, in turn, could provide insights into potential female protective factors (54). To accomplish this, we integrated task-based functional MRI (fMRI) data obtained in response to point-light displays of whole-body human motion, a well-established measure of social perception (55), and genetic data regarding median size of rare, genic CNVs (i.e., those containing one or more genes) as a measure of mutation severity, in a sex-balanced cohort of ASD and typically-developing youth (8–17 years-old; total imaging *n* = 184, males = 94, females = 90). Differences in brain response between our sex-balanced cohorts could be due to environmental factors, such as the cumulative effects of life experiences, and/or to biological factors, such as the effects of genetic mutations. The integration of genetic data would help determine to what degree genetically-mediated processes contribute to a possible etiological mechanism. Interestingly, previous research identified hypoactivation of the posterior superior temporal sulcus during biological motion perception as a “neural signature” of ASD (55). However, as with much of the ASD literature, this study was conducted with a male-predominant sample. In contrast, we found that ASD females, compared to typically-developing females, showed significant hypoactivation to biological motion in primarily sensorimotor, striatal, and frontal brain regions (Figure 1A). Furthermore, ASD females compared to ASD males had larger median size of rare, genic CNVs containing gene(s) expressed in early development of these brain regions, especially the striatum (Figure 1B), which was replicated in an independent cohort (SSC) of ASD females and males. The striatum is a subcortical structure involved in motor planning as well as cognitive processes, such as social and language functions, and may link motivation to voluntary behavior. Not only do these larger CNVs support the female protective effect model, but they also suggest that impacts to the striatum may particularly contribute to female risk for autism. Therefore, caution should be applied to making inferences about female autism based on male-predominant studies.

We proposed that the brain regions underlying the female protective effect may consist of those that not only differentiate typically-developing females from ASD females but also differentiate typically-developing females from typically-developing males, reflecting what is unique about the female perception of social stimuli. Comparing the brain response of these cohorts to biological motion, we found the female protective effect to be potentially represented by frontal and parietal regions of the salience and central executive brain networks, involved in processes such as monitoring salient stimuli, attention, working memory, and cognitive control. Although a lack of transcriptome data for important brain regions comprising these networks precluded the integration of genetic data as had been done for the female autism neural profile, important insights were still gained into the regions relating to female resilience in social perception. However, further studies are needed to elucidate the genetic and/or environmental factors affecting the differential development of these networks.

### Studies of Prenatal Sex Steroids

One of the most well-known theories for the sex bias in ASD is the “extreme male brain theory” proposed by Simon Baron-Cohen, which holds that ASD presents as an extreme version of the male brain, where individuals with ASD exhibit greater abilities in male “systemizing” as opposed to female “empathizing” characteristics (56). Consistent with this idea, Baron-Cohen proposed that fetal exposure to sex steroids, specifically fetal androgens, might contribute to the sex bias in ASD (57). A number of clinical studies, many of which are from Baron-Cohen's group, have directly investigated associations between prenatal exposure to sex steroid hormones, including androgens and estrogens, and ASD (reviewed in Table 1) (58–64). In support of this theory, Baron-Cohen's research group identified a positive correlation between fetal testosterone levels measured in amniotic fluid by amniocentesis and the development of ASD traits in typically developing male (*n* = 118) and female (*n* = 117) children at ages 6–10 years in a longitudinal cohort study (58). This significant positive relationship between fetal testosterone levels and ASD traits was also observed in typically developing toddlers aged 18–24 months (59). In another study, Baron-Cohen et al. (60) compared levels of cortisol and the sex steroids progesterone, 17α-hydroxy-progesterone, androstenedione, and testosterone, from amniotic fluid samples of males who were later diagnosed with ASD (*n* = 128) and typically developing males (*n* = 217) from the Danish Historic Birth Cohort, and found that the levels of all hormones were positively correlated with each other and that all hormones were elevated in the ASD group on “a latent generalized steroidogenic factor” ascertained by principal components analysis (60). This study provided evidence that increased steroidogenic activity may be associated with ASD.

**Table 1:** Clinical and postmortem studies of sex steroid hormones in autism spectrum disorder.

**Prenatal studies**	**Results**	**Sample type**	**Age at assessment**	**Samples size/Sex**	**Diagnosis**
Auyeung et al. (58)	• Positive association of fetal testosterone levels and higher scores on the Childhood Autism Spectrum Test (CAST) and the Child Autism Spectrum Quotient (AQ-Child) in typically developing children	Amniotic fluid	6–10 years	235 M = 118, F = 117	Typically developing children
Auyeung et al. (59)	• Positive association between fetal testosterone levels and scores on the Quantitative Checklist for Autism in Toddlers (Q-CHAT) in typically developing children. • Males had higher Q-CHAT scores indicating more “autistic traits” compared to females	Amniotic fluid	18–24 months	129 M = 66, F = 63	Typically developing children
Baron-Cohen et al. (60)	• Positive association of levels of progesterone, 17α-hydroxy-progesterone, androstenedione, testosterone, and cortisol with each other in amniotic fluid • Elevated levels of all hormones in the ASD group on a generalized latent steroidogenic factor that accounted for much of the variation in the data by principal component analysis	Amniotic fluid	Individuals born between the years 1993 and 1999 in the Danish Historic Birth Cohort	Control (*n* = 217); ASD (*n* = 128)/Males only	ICD-10 diagnosis of any ASD code
Baron-Cohen et al. (61)	• Increased levels of prenatal amniotic estradiol, estriol, and estrone were predictive of an ASD diagnosis • Elevated prenatal amniotic estradiol was the most significant predictor of ASD diagnosis in univariate logistic regression model	Amniotic fluid	Individuals born between the years 1993 and 1999 in the Danish Historic Birth Cohort	Control (*n* = 177); ASD (*n* = 98)/Males only	ICD-10 diagnosis of any ASD code
Bilder et al. (62)	• Higher estradiol and lower sex hormone binding globulin levels were associated with ASD risk in a cohort enriched for prenatal metabolic syndrome	Prenatal maternal serum (early second trimester)	Individuals born to mothers participating in the FASTER study from 1999 to 2002	Control (*n* = 19); ASD (*n* = 53)/Samples were matched by sex and birth year	ASD diagnostic billing codes; special education autism exceptionality status
**Clinical studies**	**Results**	**Sample type**	**Age at assessment**	**Samples size/sex**	**Diagnosis**
Sharpe et al. (63)	• B-lymphocytes from individuals with ASD showed less growth depression and mitochondrial upregulation in response to estradiol and dihydrotestosterone compared to unaffected siblings and unrelated controls	B-lymphocytes	Samples from the AGRE tissue bank; controls were age- and sex-matched	Unrelated controls (*n* = 11), M = 10, F = 1; Unaffected fraternal twins (*n* = 10), M = 4, F = 6; unaffected siblings (*n* = 22), M = 11, F = 11; ASD (*n* = 11), M = 10, F = 1	ASD diagnosis from AGRE
Altun et al. (64)	• Decreased serum GPER levels in the ASD group	Blood	Control = 6.12 ± 2.55 years; ASD = 5.33 ± 2.61 years	Control (*n* = 40), M = 32, F = 8; ASD (*n* = 45), M = 40, F = 5	DSM-V diagnosis of ASD
**Postmortem studies**	**Results**	**Brain regions**	**Age at tissue collection**	**Samples size/sex**	**Sample source**
Sarachana et al. (65)	• Decreased aromatase and RORA proteins in frontal cortex of ASD subjects relative to sex- and age- matched controls • Aromatase protein strongly correlates with protein levels of RORA, which transcriptionally regulates aromatase	Frontal cortex	Age- and sex-matched controls	Control (*n* = 22); ASD (*n* = 12)	Autism Tissue Program
Crider et al. (66)	• Decreased ERβ and CYP19A1 (aromatase) mRNA in ASD subjects; no change in ERα • Decreased mRNA levels of the ER co-activators: SRC-1, CBP and P/CAF in ASD subjects • Decreased ERβ and CYP19A1 protein levels in ASD subjects	Middle frontal gyrus	Control: 11.70 ± 1.584; ASD: 11.80 ± 1.609	Control (*n* = 13), M = 12, F = 1; ASD (*n* = 13), M = 13, F = 0	NICHD Brain and Tissue Bank for Developmental Disorders at the University of Maryland

*AGRE, Autism Genetic Resource Exchange; ASD, Autism spectrum disorder; CBP, CREB-binding protein; ER, Estrogen receptor; ERα, Estrogen receptor Alpha; ERβ, Estrogen receptor Beta; F, female; FASTER, First and Second Trimester Evaluation of Risk; GPER, G protein-coupled estrogen receptor; ICD-10, International Statistical Classification of Diseases and Related Health Problems, 10th revision; M, male; NICHD, National Institute of Child Health and Human Development; P/CAF, p300/CREB-binding protein-associated protein; RORA, Retinoic acid-related orphan receptor-alpha; SRC-1, steroid receptor co-activator-1*.

By analyzing the same cohort from the Danish Historic Birth Cohort of males with (*n* = 98) and without ASD (*n* = 177), Baron-Cohen et al. (61) found that elevated levels of prenatal amniotic estradiol, estriol, and estrone were significantly associated with an ASD diagnosis in univariate logistic regression analysis (61). Of the steroidogenic hormones analyzed in the previous study (60), only progesterone levels were found to be significantly associated with an ASD diagnosis (61). Interestingly, this study suggests that increased levels of estrogens are associated with an ASD diagnosis in males, though due to the limited number of females with ASD in this cohort, additional studies are needed to investigate whether this association exists for females with ASD (61). Baron-Cohen et al. (61) viewed the results of this and their previous study as complementary, in that both suggest that increased prenatal steroidogenic activity may be associated with ASD.

In line with this theory, one study found that females with congenital adrenal hyperplasia (*n* = 34), a condition caused by an enzyme deficiency that leads to an increase in fetal androgen levels, have higher scores on the Autism Spectrum Quotient compared to unaffected female relatives (*n* = 24) (67). In addition, a matched case-control study of children ages 4–17 born in Sweden from 1984 to 2007 (*n* = 23,748 ASD cases and *n* = 208,796 controls) found that there is a 59% increased risk of ASD in children of women with polycystic ovary syndrome, which leads to increased androgen levels, and a greater risk if women had both PCOS and obesity (68). Further, males with aneuploidies affecting the Y chromosome (e.g., 47, XYY; 48, XXYY) were found to be 20 times more likely to have an ASD diagnosis than males in the general population (69). In a study of 860 individuals with Klinefelter syndrome in Sweden with 86,000 population controls, Klinefelter syndrome was found to be associated with a six times higher risk of ASD as well as an increased risk of schizophrenia and ADHD (70). Another study found that 21% of individuals with Turner syndrome (45, X) (*n* = 98) met the criteria for a diagnosis of ASD, which was higher than the national rates of ASD in females in the United Kingdom (0.3%) (71). Collectively, these studies suggest that altered exposure to sex steroids may be associated with ASD, though additional research will be important to elucidate potential mechanisms.

### Post-mortem Studies of Sex Steroids

A limited number of post-mortem studies have examined the expression of genes related to sex steroid signaling in individuals with ASD (Table 1). For example, Crider et al. (66) found that levels of estrogen receptor-beta (ERβ) mRNA and protein were significantly reduced in the middle frontal gyrus of ASD subjects (*n* = 13) compared to controls (*n* = 13) (66). In addition, expression levels of coactivators of the estrogen receptor, including steroid receptor co-activator 1, CREB-binding protein, and p300/CREB-binding protein-associated protein, were lower in the middle frontal gyrus of ASD compared to control subjects (66). Another post-mortem study found that protein levels of retinoic acid-related orphan receptor-alpha (RORA), whose expression is differentially regulated by estradiol and dihydrotestosterone, as well as aromatase, the enzyme that converts testosterone into estrogen and whose expression is regulated by RORA, were significantly reduced in the frontal cortex of individuals with ASD (*n* = 12) compared to controls (*n* = 22) (65). However, additional post-mortem studies with larger samples sizes are needed.

## Functional Studies of ASD Risk Genes Identify Estrogens as Potential Modulators

**Figure 2:**
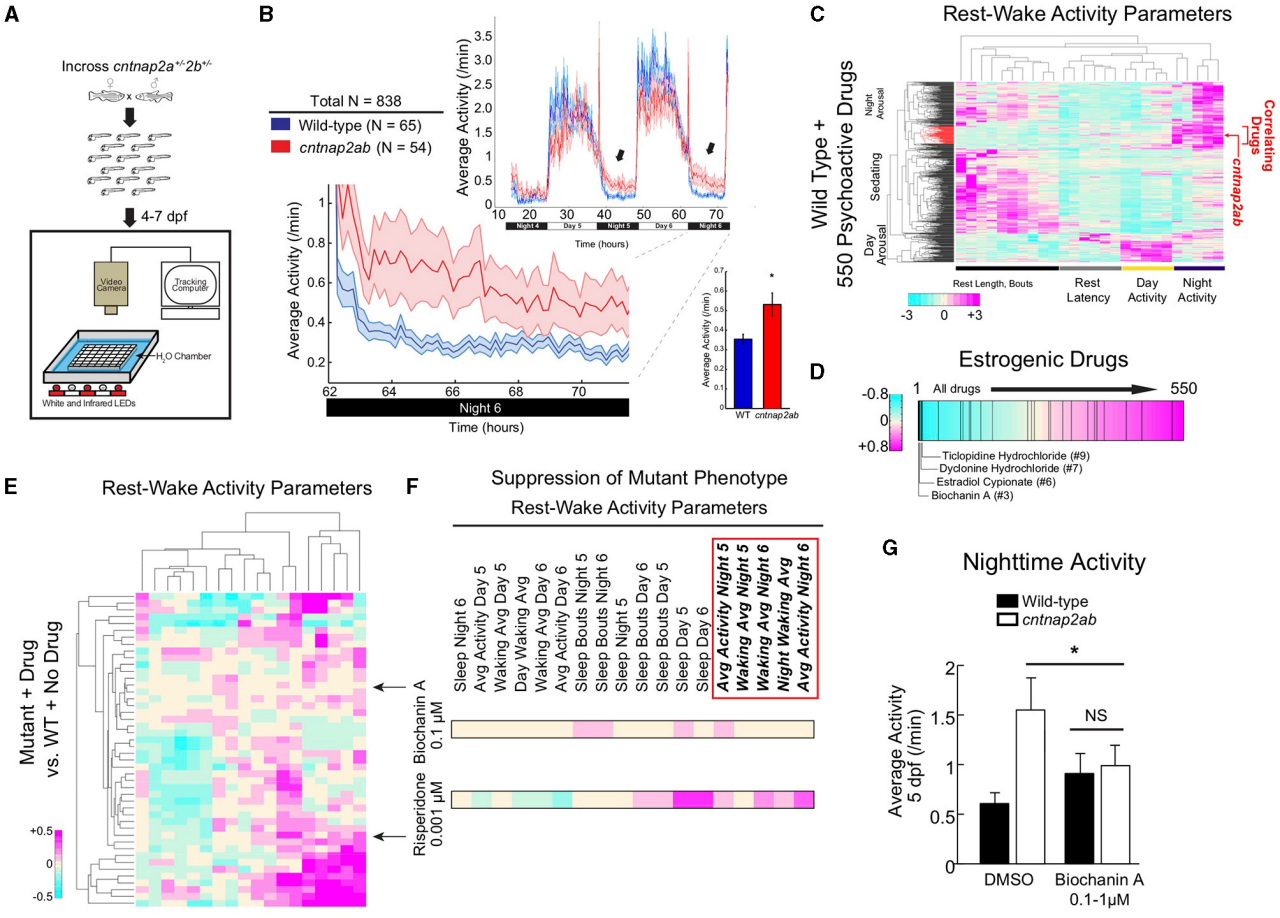
The plant-derived estrogen, biochanin A, reverses nighttime hyperactivity in zebrafish mutants of the ASD risk gene, *CNTNAP2*. **(A)** Experimental set-up. Locomotor activity of zebrafish larvae is tracked on a rest-wake cycle from 4 to 6 days post fertilization (dpf) using an automated assay (77, 78). **(B)** Locomotor activity of *cntnap*2*a*^Δ*25*/Δ*25*^*cntnap*2*b*^Δ*7*/Δ*7*^ (*cntnap2ab*, red) and wild-type (WT; blue) sibling-matched larvae is shown over 72 h. Hyperactivity in mutants worsens on successive nights (arrows). The magnified activity profile on night 6 is shown. Inset graph shows average locomotor activity of *cntnap2ab* vs. wild-type. *p = 0.00012 (one-way ANOVA, comparing all genotypes on all nights); *p* = 0.0193, 0.0236, and 0.0073, nights 4, 5, and 6, respectively. **(C)** Hierarchical clustering of the *cntnap2ab* behavioral fingerprint (red arrow) compared with the fingerprints of wild-type larvae exposed to a panel of 550 psychoactive agents from 4 to 6 days post fertilization (dpf) (77). Each rectangle in the clustergram represents the Z score, or the average value in standard deviations relative to the behavioral profiles of wild-type exposed to DMSO alone (magenta, higher than DMSO; cyan, lower than DMSO). The *cntnap2ab* profile correlates with agents that induce nighttime arousal (“Correlating Drugs”). **(D)** Rank-sorting of the anti-correlating dataset with respect to estrogenic compounds shows significant enrichment of estrogenic agents in the top ranks (*p* = 0.0003 by random permutation). Black lines indicate drugs defined as having estrogenic activity (25 compounds in total). **(E)** Hierarchical clustering of the behavioral fingerprints of *cntnap*2*a*^Δ*121*/Δ*121*^*cntnap*2*b*^*31i*/*31i*^ larvae exposed to 14 psychoactive agents at three doses each relative to the wild-type + no drug fingerprint. Each rectangle in the clustergram represents the Z score of drug-exposed mutants relative to untreated wild-type (magenta, higher than wild-type; cyan, lower than wild-type). **(F)** Magnified sections of the clustergram show relative suppression of the mutant fingerprint by biochanin A (0.1 μM) compared with risperidone (0.001 μM). The red box highlights parameters that measure nighttime activity. **(G)** Effect of the blind addition of biochanin A (0.1–1 μM) or DMSO on nighttime activity in the progeny of incrosses of *cntnap*2*a*^Δ*25*/+^*cntnap*2*b*^Δ*7*/+^ fish at 5 dpf. *p = 0.045 (two-way ANOVA, gene x dose interaction). Figure adapted and reprinted with permission from Hoffman et al. (8).

Interestingly, recent preclinical studies investigating the function of ASD risk genes in distinct model systems, including one from our group, found that estrogens might play a modulatory role in signaling pathways related to ASD and other neurodevelopmental disorders (Table 2) (8, 9, 48, 72). First, our group investigated the ASD and epilepsy risk gene, *Contactin Associated Protein-like 2* (*CNTNAP2*), and identified estrogenic compounds in an unbiased screen as a suppressor of a behavioral phenotype in zebrafish lacking the function of this gene (8). *CNTNAP2*, which encodes a cell adhesion molecule in the neurexin family, was first identified as a risk gene by a linkage study in consanguineous families from the Old Order Amish population with cortical dysplasia focal epilepsy syndrome (73). Our group utilized zebrafish to analyze the function of this gene, because they are highly tractable and amenable to large-scale small molecule screens (74, 75). We found that zebrafish mutants of *cntnap2* display selective deficits in GABAergic neurons, particularly in the forebrain, consistent with findings in mouse *Cntnap2* knockouts (76). Using a large-scale assay to quantify rest-wake activity (77), we found that zebrafish *cntnap2* mutants have a specific behavioral phenotype of nighttime hyperactivity (Figures 2A,B). To identify pharmacological compounds that suppress the *cntnap2* mutant behavioral “fingerprint,” we screened small molecules by comparing the mutant behavioral phenotype to an existing dataset of the behavioral profiles of wild-type fish exposed to 550 psychoactive compounds across multiple parameters (77) (Figure 2C). Interestingly, we found that estrogenic compounds were significantly enriched in the top ranks of small molecules that anti-correlate with, or generate the opposite profile of, the *cntnap2* behavioral “fingerprint” (Figure 2D). Further, in a screen of 14 psychoactive compounds, including those with estrogenic activity, we found that the plant-derived estrogen, biochanin A, most selectively reversed the mutant behavioral phenotype by decreasing nighttime activity with little effect on other measures of rest and activity and with greater specificity than risperidone, which is FDA-approved to treat aggression and irritability in ASD (8) (Figures 2E–G). While the mechanisms mediating the rescue by estrogens are not clear, it is possible that estrogens might act by modulating deficits in GABAergic and glutamatergic signaling in mutants. While early exposure to biochanin A did not reverse the structural GABAergic deficits (8), additional studies are needed to determine how estrogens might affect excitatory and inhibitory circuits in these mutants.

**Table 2:** Preclinical studies of estrogens in genetic models of ASD and other neurodevelopmental disorders.

**Study**	**Gene(s)**	**Model system**	**Developmental stage**	**Brain region(s)**	**Results**
Olivetti et al. (48)	*ARX*	Mouse	Early postnatal and Adult	Cortex	• Early postnatal exposure to estradiol prevented spasms in infancy and seizures in adult mutants • Estradiol reversed deficits in neuropeptide Y-positive cortical interneuron and striatal cholinergic interneuron populations
Hoffman et al. (8)	*CNTNAP2*	Zebrafish	Larval (4–6 days post fertilization)	N/A	• Estrogens, including the plant-derived estrogen, biochanin A, and β-estradiol-17-cypionate, identified in a drug screen, suppressed a behavioral phenotype of nighttime hyperactivity in zebrafish *cntnap2* mutants
Erli et al. (72)	*DISC1*	Rat	Embryonic	Cortical neuronal culture	• Acute 17-β-estradiol rescued dendritic spine deficits and reduced DISC1 aggregation in neurons overexpressing wild-type or mutant DISC1 • Acute 17-β-estradiol rescued dendritic spine deficits due to DISC1 knockdown • Chronic 17-β-estradiol rescued dendritic spine deficits and reduced DISC1 aggregation in neurons expressing mutant DISC1 • Chronic 17-β-estradiol increased the number of spines positive for pre- and post-synaptic markers in control and mutant DISC1-expressing neurons
Willsey et al. (9)	*ADNP; ANK2;* *ARID1B;* *CHD2;* *CHD8;* *DYRK1A;* *NRXN1;* *SCN2A;* *SYNGAP1;* *POGZ*	Frog (*Xenopus tropicalis*)	Tadpole	Telencephalon	• An estrogenic agonist, estramustine, identified in a drug screen reversed the phenotype of increased proliferation in the *Xenopus* telencephalon induced by chemical disruption of the ASD risk gene, *DYRK1A* • Disruption of estrogenic signaling led to a reduction in telencephalon size in *Xenopus* • 17-β-estradiol reversed increased proliferation in NPCs derived from human CRISPRi cell lines disrupting the ASD risk genes *DYRK1A, NRXN1*, or *ADNP*

*ASD, Autism spectrum disorder; ARX, Aristaless-Related Homeobox; ADNP, Activity-dependent neuroprotective protein; ANK2, Ankyrin 2; ARID1B, AT-Rich Interaction Domain 1B; CHD2, Chromodomain Helicase DNA Binding Protein 2; CHD8, Chromodomain-helicase-DNA-binding protein 8; CNTNAP2, Contactin-associated protein-like 2; CRISPRi, clustered regularly interspaced short palindromic repeats interference; DISC1, Disrupted-in-schizophrenia 1; DYRK1A, Dual specificity tyrosine phosphorylation regulated kinase 1A; NPCs, neural progenitor cells; NRXN1, Neurexin 1; POGZ, Pogo Transposable Element Derived with ZNF Domain; SCN2A, Sodium Voltage-Gated Channel Alpha Subunit 2; SYNGAP1, Synaptic Ras GTPase-activating protein 1*.

Remarkably, another recent study also found that estrogens might act as modulators of cell proliferation phenotypes across multiple ASD risk genes (9). Willsey et al. (9) performed an *in vivo* CRISPR screen in *Xenopus tropicalis* targeting 10 high confidence ASD risk genes. By capitalizing on a unique feature of *Xenopus*, in which it is possible to generate unilateral mutants by introducing CRISPRs into one cell at the two-cell stage and compare phenotypes on either side of the midline at later stages, the authors found that disruption of high confidence ASD risk genes resulted in altered telencephalon size as well as an increased ratio of neural progenitor cells to neurons in this region (9). Disruption of ASD risk genes in human induced pluripotent stem cell (iPSC)-derived neural progenitor cells (NPCs) using CRISPR interference (CRISPRi) also led to an increase in the proportion of proliferative cells, which together with the findings in *Xenopus*, suggests that dysregulated neurogenesis may represent a convergent pathway linking these genes (9). Intriguingly, in a screen of 133 FDA-approved oncology drugs to identify compounds that reverse altered proliferation in the *Xenopus* telencephalon (induced by chemical disruption of the ASD risk gene, *DYRK1A*), 3 of the top 17 compounds identified as modulating this phenotype affect estrogen signaling. These include estramustine, an estrogenic agonist, which rescued the phenotype by decreasing the number of neural progenitor cells relative to neurons, along with raloxifene and fulvestrant, which are estrogenic inhibitors that led to an increase in the neural progenitor cell to neuron ratio. Estradiol also had similar effects in rescuing an altered proportion of proliferative cells in human NPCs following disruption of ASD risk genes. This study found that estrogen signaling is involved in regulating neurogenesis in the telencephalon, and identified Sonic hedgehog (SHH) signaling as a potential pathway by which estrogens may affect neurogenesis. Importantly, this study provides support for the idea that estrogens may play a modulatory role in the developing brain by conferring resilience against the negative effects of ASD risk gene disruption, possibly through its effects on neurogenesis (9).

In addition, there is evidence that exposure to estradiol during the early postnatal period reduced seizures in a mouse model of the X-linked epilepsy gene, *ARX* (48), and that estradiol exposure reversed the reduction in dendritic spine density associated with overexpression or knockdown of the schizophrenia-associated gene, *DISC1*, in rat cortical neuronal cultures (72) (Table 2). While the study in mice was performed at doses comparable to physiological levels of estradiol in fetal plasma (48), translating the concentrations of estradiol and other estrogenic compounds from zebrafish, *Xenopus*, and *in vitro* studies to physiological levels in mammals represents a challenge, and additional research is needed to establish dose equivalency. Nonetheless, these studies suggest that estrogens may function as modulators of signaling pathways downstream of risk genes associated with ASD and other neurodevelopmental disorders.

## Estrogen Signaling Pathways With Relevance to ASD

These preclinical studies raise important questions regarding how estrogens affect basic mechanisms of brain development, though the precise mechanisms by which they might influence signaling pathways relevant to ASD have yet to be elucidated. In the following sections, we consider known cellular and molecular effects of estrogens in the brain that might overlap with signaling pathways and sexual differential processes implicated in ASD. These pathways might represent potential avenues for future investigation.

### Sexual Differentiation of the Mammalian Brain

The gonadal sex steroid hormones, estradiol and testosterone, affect sexual differentiation of the mammalian brain. To place the preclinical studies discussed in the previous section in context, here we briefly review these mechanisms, though for a more extensive discussion, see excellent reviews by McCarthy (11) and Ferri et al. (47). During an *in utero* organizational period, fetal testosterone is produced by the testes in males, controlling masculinization of the body and brain, while an absence of hormones is associated with feminization [reviewed in (47)]. Specifically, the aromatization hypothesis, based on studies of perinatal rats, holds that testicularly derived fetal testosterone is aromatized locally to estradiol in the male brain. Estradiol then induces masculinization in males, while the lack of exposure to androgens, and its aromatized product, estradiol, leads to female brain development [reviewed in (11)]. Estradiol produced by the placenta results in high maternal levels of estradiol, which are sequestered by α-fetoprotein, a binding globulin that prevents maternal estradiol from masculinizing the brain (11). α-fetoprotein further protects female fetuses from masculinization; therefore, the impact of estrogens is differential in males vs. females (11). However, in addition to being exposed to maternal and gonadally-derived estradiol, fetuses and newborns are exposed to estradiol synthesized locally in the brain, which can exert effects independent of sexual differentiation (11). Overall, the impact of estrogens is restricted to a specific developmental window (mid-to-late gestation in primates), and includes both processes of sexual differentiation and reproduction-independent processes (11).

### Genomic and Non-genomic Effects of Estrogens

Estrogens exert their effects through genomic and non-genomic modes of action. In the classical genomic mechanism, estradiol, the bioactive form of estrogen, binds to estrogen receptor-alpha (ERα) and estrogen receptor-beta (ERβ) in the cytoplasm, causing the estrogen receptors to dimerize and translocate to the nucleus. The nuclear estradiol-estrogen receptor complex binds directly to estrogen response elements in the promoters of target genes and acts as a ligand-dependent transcription factor, regulating the expression of hundreds of genes (79). Estrogens also exert effects through non-genomic mechanisms, which involve the binding of estradiol to membrane-bound estrogen receptors, resulting in the rapid activation of intracellular signaling cascades, including mitogen activated protein kinase/extracellular signal-regulated kinase (MAPK/ERK), phosphatidylinositol 3-kinase/protein kinase B/mTOR (PI3K/Akt/mTOR), and cAMP response element-binding protein (CREB)/brain derived neurotrophic factor (BDNF) [reviewed in (80)]. Estrogen binding to G-protein-coupled estrogen receptor (GPER) has been shown to initiate the rapid actions of estrogens in *in vitro* studies (81–83), though the precise mechanisms by which estrogens induce rapid non-genomic effects *in vivo* are not well-understood and may involve ERα and ERβ acting in a “non-classical” manner (84, 85). Estrogen signaling via these pathways has been shown to function in synaptogenesis (86–88) and play a neuroprotective role (discussed below). For example, acute administration of 17-β-estradiol was found to activate ERK1/2 in cultured embryonic rat cortical neurons and lead to a transient increase in dendritic spines (88). 17-β-estradiol was also found to activate phosphorylation of ERK and Akt, as well as the N-methyl-D-aspartate receptor subtype 2B (NR2B) subunit of NMDA receptors, in rat cortical synaptoneurosomes, providing evidence that estradiol acts directly at cortical synapses and regulates intracellular signaling pathways (86). There is also evidence for “crosstalk” between the ERK/MAPK and PI3K/Akt pathways downstream of estrogen signaling. For example, treatment with PI3K/Akt inhibitors blocked estradiol-mediated phosphorylation of both ERK and Akt in rat cortical synaptoneurosomes (86). In addition, 17-β-estradiol was shown to rapidly induce both MAPK and PI3K signaling *in vitro* in embryonic rat cortical neurons (89). Interestingly, a recent study examined mice lacking aromatase in forebrain neurons, which have decreased regional estradiol, and found that they display decreased MAPK/ERK and PI3K/Akt signaling, as well as reduced CREB/BDNF in the cerebral cortex and hippocampus (90). These mice also exhibit reduced synaptic density in the forebrain, decreased long-term potentiation (LTP) amplitude in hippocampal slices, as well as significant deficits in hippocampal-dependent spatial reference memory, recognition memory, and contextual fear memory. Restoring 17-β-estradiol acutely rescued the LTP deficit, while treatment with an ERK inhibitor prevented the rescue, providing evidence that estrogen signaling via ERK is important for learning and memory (90).

Interestingly, there are several lines of evidence implicating these intracellular signaling pathways in ASD, independent of their role in estrogen signaling. For example, as discussed above, dysregulation of PI3K/Akt/mTOR signaling has been described in rare monogenic disorders associated with ASD, including tuberous sclerosis complex, Fragile X syndrome, and neurofibromatosis [reviewed in (19)]. In addition, alterations in CREB and cAMP signaling have been identified in Fragile X syndrome (91, 92), and an allosteric inhibitor of an enzyme that degrades cAMP was recently found to significantly improve cognition in adult males with Fragile X syndrome in a randomized, placebo-controlled phase 2 trial (93). Moreover, a recent preclinical study found that male, but not female, mice with the 16p11.2 hemideletion have deficits in reward learning and increased ERK1 activation in the striatum, suggesting that regional alterations in ERK signaling might modulate sex-differential behaviors in the context of an ASD-associated CNV (94). Studies aimed at elucidating the connection between these intracellular signaling pathways and ASD, as well as their possible modulation by estrogens, represent important avenues for further investigation.

### Estrogen Effects on Excitatory and Inhibitory Signaling

Estrogens have been shown to play a role in GABAergic neuron development as well as protect against glutamate-induced neurotoxicity. Estradiol influences the shift from excitatory to inhibitory GABAergic signaling in the developing brain. That is, GABA_A_ receptors, which are ligand-gated chloride channels, exhibit depolarizing effects during the neonatal period and classical hyperpolarizing signaling at later stages. This shift from excitation to inhibition is mediated by changes in chloride co-transporter expression and activity, which alter the reversal potential for chloride [see excellent review by McCarthy (11)]. Estradiol has been shown to enhance the depolarizing effects of GABA in developing hypothalamic and hippocampal neurons (11, 95, 96). However, estradiol-promoted enhancement of the excitatory GABA effect may result in a lower threshold for excitotoxicity (11). Therefore, estradiol seems to have opposite effects on GABAergic and glutamatergic signaling in the developing brain, increasing GABA-induced excitotoxicity, while protecting against glutamate-induced excitotoxicity (11).

The neuroprotective effects of estrogen have been studied most extensively in the context of hypoxia and ischemia in the adult brain [see reviews by (97, 98)], yet less is known about its neuroprotective role in the developing brain (11). Interestingly, estrogen signaling via the intracellular pathways discussed above has been shown to mediate its neuroprotective effects. For example, estrogens were found to induce neuroprotective effects against glutamate-induced excitotoxicity in embryonic rat primary cortical neurons by activation of ERK/MAPK signaling (99). PI3K/Akt signaling has also been shown to mediate estrogen-induced neuroprotection. For example, pretreatment of cultured rat fetal cortical neurons with estradiol for 24 h prior to glutamate exposure significantly reduced glutamate-induced neurotoxicity, while exposure to a PI3K inhibitor significantly attenuated the neuroprotective effect of estradiol (100). In a follow-up study, estradiol was shown to induce phosphorylation of CREB and upregulate the anti-apoptotic protein, Bcl-2, in a PI3K/Akt-dependent manner, suggesting that estradiol exerts neuroprotective and anti-apoptotic effects via activation of the PI3K/Akt pathway (101).

Estrogens have also been shown to affect signaling and/or induce neuroprotective effects in other neural cell types that have been implicated in ASD, including oxytocin neurons (102, 103), dopaminergic neurons (104–106), as well as astrocytes and microglia (107–109), though a detailed discussion of these mechanisms is beyond the scope of this review. While estrogens have broad neurodevelopmental effects on multiple neural cell types, additional investigations are needed to determine which of these mechanisms might be most relevant to ASD pathophysiology.

### Estrogen Effects on Synapse Formation and Function

As discussed above, estrogens have been shown to function in synaptogenesis (87, 88, 110). In particular, estradiol induces rapid effects at the synapse, including the activation of metabotropic glutamate receptors (mGluRs) mediated by ERα and ERβ, intracellular signaling cascades, actin polymerization, and local protein synthesis, which together may function in the “fine-tuning” of neural circuits (110). Interestingly, estrogens have been shown to play a critical role in synaptic plasticity and memory consolidation in the hippocampus [see excellent review by Frick (84)]. Studies have shown that infusion of 17-β-estradiol into the dorsal hippocampus or dorsal third ventricle enhances object recognition memory consolidation in young ovariectomized female mice, such that mice spend significantly more time with a novel vs. a familiar object following estradiol infusion [(111); reviewed in (84)]. Estradiol-induced memory consolidation requires ERK phosphorylation, which is activated by interactions between ERα and ERβ and metabotropic glutamate receptor 1 (mGluR1) (112, 113). Inhibitors of MAPK, PI3K, and mTOR (rapamycin) have been shown to block ERK phosphorylation and estradiol-induced hippocampal memory consolidation, indicating that the rapid activation of these signaling pathways is involved in this process (114–116). Estradiol-induced ERK activation also leads to phosphorylation of CREB, as well as increased histone H3 acetylation (117). Blocking histone acetylation also prevents estradiol-induced memory consolidation, demonstrating that epigenetic effects downstream of estrogen signaling play an important role in this process as well (118). All together, these studies highlight a central role for estrogens in shaping synaptic function and neural circuits.

## Discussion

In the past 10 years, there has been remarkable progress in ASD risk gene discovery, enabling the identification of common mechanistic pathways involving these genes, including the regulation of gene expression, synaptogenesis, and excitatory-inhibitory imbalance, leading to insights into basic biological processes that may be disrupted in ASD. It has long been recognized that ASD has a strong male bias, even when taking into account ascertainment bias, which may contribute to a decreased tendency to diagnose ASD in females, yet the basis for the male predominance is not well-understood. At a genetics level, there is evidence for a “female protective effect,” such that females are more likely to carry mutations of greater impact, suggesting that females have a higher liability threshold for ASD diagnosis and/or that males have a lower liability threshold (7). While the etiology of the female protective effect is not well-understood, recent studies using genetics, transcriptomics, imaging genomics, as well as animal models are beginning to shed light on potential relevant mechanisms.

First, genetics studies to date have not identified a unifying genetic mechanism that accounts for resilience in females and/or risk in males (5, 7, 50), suggesting that the sex bias may not be driven by primarily genetic factors. Consistent with this finding, the first transcriptomic study of sex-differential processes in ASD (7) found that genes with dysregulated expression patterns in post-mortem brains of individuals with ASD, but not ASD risk genes themselves, show sex-differential expression patterns. This intriguing finding suggests that factors downstream of ASD risk genes likely interact with sex-differential processes to contribute to the sex bias in ASD. Second, a recent imaging genomics study from our group found that females with ASD show decreased activation of sensorimotor, striatal, and frontal brain regions in response to biological motion and have larger rare, genic CNVs containing genes expressed in these brain regions, particularly the striatum (54). In addition to finding a novel convergence in genetics and imaging data, this study further indicates that brain regions responsible for differences in the perception of social stimuli in typically developing females and males may also contribute to the female protective effect in ASD (54). Third, consistent with the “extreme male brain” theory, studies of prenatal exposure to sex steroid hormones in humans indicate that increased steroidogenic activity might be associated with increased ASD risk (60, 61) [reviewed in (7, 47)]. However, the mechanisms by which differential exposure to androgens and/or estrogens might contribute to risk or resiliency are not well-understood. Additional clinical studies with larger sample sizes of males and females will be important for further clarifying this association.

Interestingly, recent preclinical studies from our group and others identified estrogenic compounds as potential suppressors of ASD gene-associated cellular or behavioral phenotypes in unbiased *in vivo* pharmacological screens in zebrafish and *Xenopus* (8, 9), and found that estrogens rescue seizures in a genetic mouse model of epilepsy (48) and cellular phenotypes associated with *in vitro* dysregulation of *DISC1* (72). While these investigations raise important questions regarding how estrogens might affect basic mechanisms of brain development, translating findings from animal and *in vitro* systems to humans presents various challenges. On the one hand, estrogens were found to suppress abnormal cellular and behavioral phenotypes associated with risk gene loss of function in preclinical studies (8, 9, 48, 72). At the same time, as discussed above, studies of prenatal exposure to sex steroids in humans found that increased steroidogenic activity is associated with ASD risk (60, 61). One possible interpretation is that the preclinical studies reflect the modulatory effects of estrogens on basic neurodevelopmental processes independent of their role in sexual differentiation. That is, estrogens may contribute to resilience during brain development by modulating signaling pathways, cellular phenotypes, and/or neural circuits that are dysregulated downstream of ASD risk genes. For example, our group hypothesized that estrogenic compounds may act by modulating GABAergic and glutamatergic signaling deficits in zebrafish *cntnap2* mutants (8). In addition, Willsey et al. (9) found that estrogens may affect neurogenesis by regulating the expression of genes in the SHH pathway. Moreover, identifying equivalent developmental time points across different systems further complicates directly translating findings from preclinical to clinical studies. Future analyses aimed at investigating the mechanisms underlying the effects of estrogens in preclinical studies, as well as the functions of estrogens and androgens in mammalian brain development and sexual differentiation will be essential for resolving these findings.

**Figure 3:**
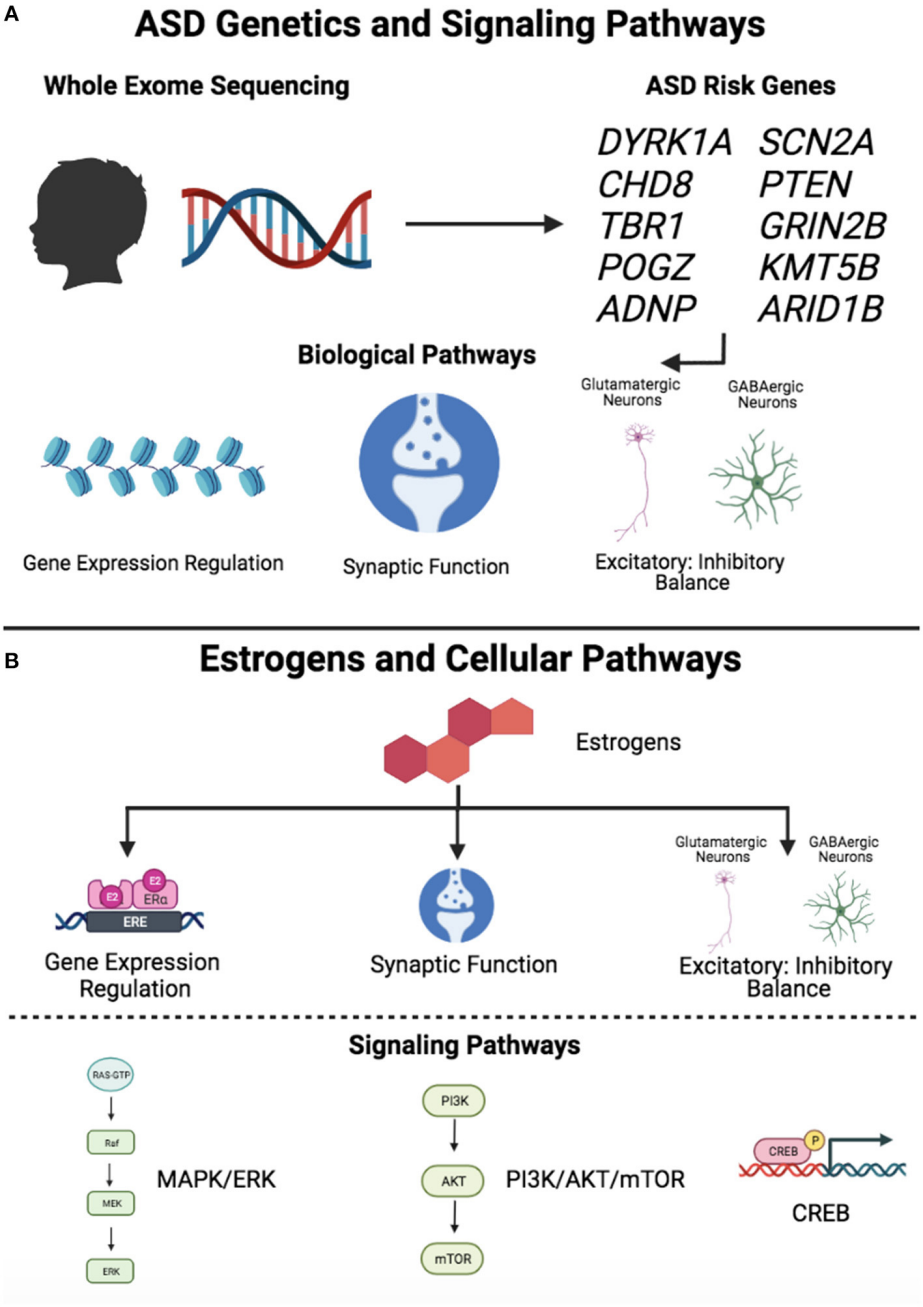
Signaling pathways downstream of ASD risk genes and estrogens. **(A)** ASD genetics and signaling pathways. Whole exome sequencing studies have led to the identification of high confidence ASD risk genes, which converge on common biological pathways, including gene expression regulation, synaptic function, and excitatory-inhibitory balance. **(B)** Estrogens and cellular pathways. Estrogens have been shown to affect several biological pathways, including gene expression regulation, synaptic function, and excitatory-inhibitory balance. Estrogens exert effects through intracellular signaling pathways, including MAPK/ERK, PI3K/Akt/mTOR, and CREB. Figure created with BioRender.com. “Gene Expression Regulation” and “PI3K/AKT/mTOR” symbols (Estrogens and Cellular Pathways, lower panel) adapted from “Estrogen Receptor Signaling” and “KRAS Signaling Pathways,” respectively, by BioRender.com (2021). Retrieved from https://app.biorender.com/biorender-templates.

While additional investigations are needed to determine the extent to which estrogens might modulate brain development with relevance to the sex bias in ASD, it is interesting to observe that there are multiple pathways downstream of estrogen signaling that intersect with biological processes implicated by ASD risk genes (Figure 3). First, estrogens have been shown to affect synaptogenesis and induce neuroprotective effects via the PI3K/Akt/mTOR, cAMP/CREB, and ERK/MAPK intracellular signaling pathways (86, 88, 90, 99–101), which have been independently implicated in ASD. Studies examining the interactions between estrogens and these pathways in the context of ASD gene-associated preclinical models are likely to be highly informative. Second, estrogens affect GABAergic neuron development and protect against glutamate-induced toxicity (11), suggesting that they may contribute to excitatory-inhibitory balance, which has been proposed as a central mechanistic pathway in ASD and other neurodevelopmental disorders (44). Third, estrogens affect synaptogenesis in developing neurons (90, 110) and play a clear role in learning and memory by promoting synaptogenesis in the adult hippocampus (84). Given that synaptic function has been identified as a key pathway in ASD, investigating the molecular mechanisms by which estrogens affect synaptogenesis and circuitry in the developing brain is likely to be important for gaining a greater understanding of their potential role as modulators. Emerging research into the roles of sex steroids in the developing brain along with studies integrating genetics, transcriptomics, and neuroimaging are likely to inform our understanding of sex differential biology in ASD. In particular, interactions between pathways implicated by ASD risk genes and the neurodevelopmental effects of estrogens may represent important avenues for future evaluation.

## Search Strategy and Selection Criteria

We searched PubMed for articles in all year ranges with multiple combinations of search terms including, “autism spectrum disorder,” “estrogen,” “estradiol,” “brain,” “genetics,” “female protective effect,” “sex differential processes,” “neuroprotection,” “MAPK,” “ERK,” “PI3K,” “Akt,” “mTOR,” “CREB,” “cAMP,” “GABA,” “glutamate,” “synapse.” Articles were selected based on relevance to topics covered in this review.

## Author Contributions

KE, AG, and EH contributed to the conceptualization, literature review, writing of this article, and approved it for publication. All authors contributed to the article and approved the submitted version.

## Funding

KE was supported by the Autism Science Foundation, Yale College Rosenfeld Science Scholars Fellowship, Yale College First-Year Summer Research Fellowship in the Sciences & Engineering, Sherwood E. Silliman Fellowship, and the Silliman College Richter Fellowship. AG was supported by NIH R01MH100028, NIH R03HD100771, Simons Foundation, and the O'Sullivan Foundation. EH was supported by NIH R01MH116002, Binational Science Foundation, Kavli Foundation, National Genetics Foundation, Simons Foundation, Spector Fund, and the Swebilius Foundation.

## Author Disclaimer

The content of this report is solely the responsibility of the authors and does not necessarily represent the official views of the National Institutes of Health.

## Conflict of Interest

The authors declare that the research was conducted in the absence of any commercial or financial relationships that could be construed as a potential conflict of interest.

## Publisher's Note

All claims expressed in this article are solely those of the authors and do not necessarily represent those of their affiliated organizations, or those of the publisher, the editors and the reviewers. Any product that may be evaluated in this article, or claim that may be made by its manufacturer, is not guaranteed or endorsed by the publisher.

## Abbreviations

ASD: autism spectrum disorder
ER: estrogen receptor
LoF: loss of function
MAPK/ERK: mitogen activated protein kinase/extracellular signal-regulated kinase
PI3K/Akt/mTOR: phosphatidylinositol 3-kinase/protein kinase B/mechanistic target of rapamycin
cAMP: cyclic adenosine monophosphate
CREB: cAMP response element-binding protein
GABA: gamma aminobutyric acid
NPC: neural progenitor cell.

## References

[1] American Psychiatric Association. Diagnostic and Statistical Manual of Mental Disorders (DSM-5^®^). Washington, DC: American Psychiatric Pub (2013).

[2] Maenner MJ Shaw KA Baio J. Prevalence of autism spectrum disorder among children aged 8 years—autism and developmental disabilities monitoring network, 11 sites, United States, 2016. MMWR Surveillance Summaries. (2020) 69:1. 10.15585/mmwr.ss6904a1 32214087 PMC7119644

[3] De Rubeis S He X Goldberg AP Poultney CS Samocha K Cicek AE. Synaptic, transcriptional and chromatin genes disrupted in autism. Nature. (2014) 515:209–15. 10.1038/nature13772 25363760 PMC4402723

[4] Iossifov I O'Roak BJ Sanders SJ Ronemus M Krumm N Levy D. The contribution of de novo coding mutations to autism spectrum disorder. Nature. (2014) 515:216–21. 10.1038/nature13908 25363768 PMC4313871

[5] Sanders SJ He X Willsey AJ Ercan-Sencicek AG Samocha KE Cicek AE. Insights into autism spectrum disorder genomic architecture and biology from 71 risk loci. Neuron. (2015) 87:1215–33. 10.1016/j.neuron.2015.09.016 26402605 PMC4624267

[6] Satterstrom FK Kosmicki JA Wang J Breen MS De Rubeis S An JY. Large-scale exome sequencing study implicates both developmental and functional changes in the neurobiology of autism. Cell. (2020) 180:568–84.e523. 10.1016/j.cell.2019.12.036 31981491 PMC7250485

[7] Werling DM. The role of sex-differential biology in risk for autism spectrum disorder. Biol Sex Differ. (2016) 7:58. 10.1186/s13293-016-0112-8 27891212 PMC5112643

[8] Hoffman EJ Turner KJ Fernandez JM Cifuentes D Ghosh M Ijaz S. Estrogens suppress a behavioral phenotype in zebrafish mutants of the autism risk gene, CNTNAP2. Neuron. (2016) 89:725–33. 10.1016/j.neuron.2015.12.039 26833134 PMC4766582

[9] Willsey HR Exner CR Xu Y Everitt A Sun N Wang B. Parallel *in vivo* analysis of large-effect autism genes implicates cortical neurogenesis and estrogen in risk and resilience. Neuron. (2021) 109:788–804.e8. 10.1016/j.neuron.2021.01.002 33497602 PMC8132462

[10] Martini M Corces VG Rissman EF. Mini-review: epigenetic mechanisms that promote transgenerational actions of endocrine disrupting chemicals: applications to behavioral neuroendocrinology. Horm Behav. (2020) 119:104677. 10.1016/j.yhbeh.2020.104677 31927019 PMC9942829

[11] McCarthy MM. Estradiol and the developing brain. Physiol Rev. (2008) 88:91–134. 10.1152/physrev.00010.2007 18195084 PMC2754262

[12] Bailey A Le Couteur A Gottesman I Bolton P Simonoff E Yuzda E. Autism as a strongly genetic disorder: evidence from a British twin study. Psychol Med. (1995) 25:63–77. 10.1017/S0033291700028099 7792363

[13] Constantino JN Todorov A Hilton C Law P Zhang Y Molloy E. Autism recurrence in half siblings: strong support for genetic mechanisms of transmission in ASD. Mol Psychiatry. (2013) 18:137–8. 10.1038/mp.2012.9 22371046

[14] Ozonoff S Young GS Carter A Messinger D Yirmiya N Zwaigenbaum L. Recurrence risk for autism spectrum disorders: a Baby Siblings Research Consortium study. Pediatrics. (2011) 128:e488–495. 10.1542/peds.2010-2825 21844053 PMC3164092

[15] Ritvo ER Freeman BJ Mason-Brothers A Mo A Ritvo AM. Concordance for the syndrome of autism in 40 pairs of afflicted twins. Am J Psychiatry. (1985) 142:74–7. 10.1176/ajp.142.1.74 4038442

[16] Rosenberg RE Law JK Yenokyan G McGready J Kaufmann WE Law PA. Characteristics and concordance of autism spectrum disorders among 277 twin pairs. Arch Pediatr Adolesc Med. (2009) 163:907–14. 10.1001/archpediatrics.2009.98 19805709

[17] Sandin S Lichtenstein P Kuja-Halkola R Larsson H Hultman CM Reichenberg A. The familial risk of autism. JAMA. (2014) 311:1770–7. 10.1001/jama.2014.4144 24794370 PMC4381277

[18] Steffenburg S Gillberg C Hellgren L Andersson L Gillberg IC Jakobsson G. A twin study of autism in Denmark, Finland, Iceland, Norway and Sweden. J Child Psychol Psychiatry. (1989) 30:405–16. 10.1111/j.1469-7610.1989.tb00254.x 2745591

[19] Winden KD Ebrahimi-Fakhari D Sahin M. Abnormal mTOR Activation in Autism. Annu Rev Neurosci. (2018) 41:1–23. 10.1146/annurev-neuro-080317-061747 29490194

[20] Autism Genome Project C Szatmari P Paterson AD Zwaigenbaum L Roberts W Brian J. Mapping autism risk loci using genetic linkage and chromosomal rearrangements. Nat Genet. (2007) 39:319–28. 10.1038/ng1985 17322880 PMC4867008

[21] Feng J Schroer R Yan J Song W Yang C Bockholt A. High frequency of neurexin 1beta signal peptide structural variants in patients with autism. Neurosci Lett. (2006) 409:10–3. 10.1016/j.neulet.2006.08.017 17034946

[22] Glessner JT Wang K Cai G Korvatska O Kim CE Wood S. Autism genome-wide copy number variation reveals ubiquitin and neuronal genes. Nature. (2009) 459:569–73. 10.1038/nature07953 19404257 PMC2925224

[23] Jamain S Quach H Betancur C Rastam M Colineaux C Gillberg IC. Mutations of the X-linked genes encoding neuroligins NLGN3 and NLGN4 are associated with autism. Nat Genet. (2003) 34:27–9. 10.1038/ng1136 12669065 PMC1925054

[24] Marshall CR Noor A Vincent JB Lionel AC Feuk L Skaug J. Structural variation of chromosomes in autism spectrum disorder. Am J Hum Genet. (2008) 82:477–88. 10.1016/j.ajhg.2007.12.009 18252227 PMC2426913

[25] Durand CM Betancur C Boeckers TM Bockmann J Chaste P Fauchereau F. Mutations in the gene encoding the synaptic scaffolding protein SHANK3 are associated with autism spectrum disorders. Nat Genet. (2007) 39:25–7. 10.1038/ng1933 17173049 PMC2082049

[26] Moessner R Marshall CR Sutcliffe JS Skaug J Pinto D Vincent J. Contribution of SHANK3 mutations to autism spectrum disorder. Am J Hum Genet. (2007) 81:1289–97. 10.1086/522590 17999366 PMC2276348

[27] Anney R Klei L Pinto D Almeida J Bacchelli E Baird G. Individual common variants exert weak effects on the risk for autism spectrum disorders. Hum Mol Genet. (2012) 21:4781–92. 10.1093/hmg/dds301 22843504 PMC3471395

[28] Gaugler T Klei L Sanders SJ Bodea CA Goldberg AP Lee AB. Most genetic risk for autism resides with common variation. Nat Genet. (2014) 46:881–5. 10.1038/ng.3039 25038753 PMC4137411

[29] Klei L Sanders SJ Murtha MT Hus V Lowe JK Willsey AJ. Common genetic variants, acting additively, are a major source of risk for autism. Mol Autism. (2012) 3:9. 10.1186/2040-2392-3-9 23067556 PMC3579743

[30] Sanders SJ Murtha MT Gupta AR Murdoch JD Raubeson MJ Willsey AJ. *De novo* mutations revealed by whole-exome sequencing are strongly associated with autism. Nature. (2012) 485:237–41. 10.1038/nature10945 22495306 PMC3667984

[31] Sebat J Lakshmi B Malhotra D Troge J Lese-Martin C Walsh T. Strong association of *de novo* copy number mutations with autism. Science. (2007) 316:445–9. 10.1126/science.1138659 17363630 PMC2993504

[32] Pinto D Delaby E Merico D Barbosa M Merikangas A Klei L. Convergence of genes and cellular pathways dysregulated in autism spectrum disorders. Am J Hum Genet. (2014) 94:677–94. 10.1016/j.ajhg.2014.03.018 24768552 PMC4067558

[33] Sanders SJ Ercan-Sencicek AG Hus V Luo R Murtha MT Moreno-De-Luca D. Multiple recurrent *de novo* CNVs, including duplications of the 7q11.23 Williams syndrome region, are strongly associated with autism. Neuron. (2011) 70:863–85. 10.1016/j.neuron.2011.05.002 21658581 PMC3939065

[34] Bucan M Abrahams BS Wang K Glessner JT Herman EI Sonnenblick LI. Genome-wide analyses of exonic copy number variants in a family-based study point to novel autism susceptibility genes. PLoS Genet. (2009) 5:e1000536. 10.1371/journal.pgen.1000536 19557195 PMC2695001

[35] Christian SL Brune CW Sudi J Kumar RA Liu S Karamohamed S. Novel submicroscopic chromosomal abnormalities detected in autism spectrum disorder. Biol Psychiatry. (2008) 63:1111–7. 10.1016/j.biopsych.2008.01.009 18374305 PMC2440346

[36] Pinto D Pagnamenta AT Klei L Anney R Merico D Regan R. Functional impact of global rare copy number variation in autism spectrum disorders. Nature. (2010) 466:368–72. 10.1038/nature09146 20531469 PMC3021798

[37] Neale BM Kou Y Liu L Ma'Ayan A Samocha KE Sabo A. Patterns and rates of exonic de novo mutations in autism spectrum disorders. Nature. (2012) 485:242–5. 10.1038/nature11011 22495311 PMC3613847

[38] O'Roak BJ Vives L Girirajan S Karakoc E Krumm N Coe BP. Sporadic autism exomes reveal a highly interconnected protein network of *de novo* mutations. Nature. (2012) 485:246–50. 10.1038/nature10989 22495309 PMC3350576

[39] SFARI Gene. SFARI Gene, pp (2021). Available online at: https://gene.sfari.org/ (accessed August 16, 2021).

[40] Willsey AJ Sanders SJ Li M Dong S Tebbenkamp AT Muhle RA. Coexpression networks implicate human midfetal deep cortical projection neurons in the pathogenesis of autism. Cell. (2013) 155:997–1007. 10.1016/j.cell.2013.10.020 24267886 PMC3995413

[41] Kang HJ Kawasawa YI Cheng F Zhu Y Xu X Li M. Spatio-temporal transcriptome of the human brain. Nature. (2011) 478:483–9. 10.1038/nature10523 22031440 PMC3566780

[42] Zhang B Horvath S. A general framework for weighted gene co-expression network analysis. Stat Appl Genet Mol Biol. (2005) 4:Article17. 10.2202/1544-6115.1128 16646834

[43] Parikshak NN Luo R Zhang A Won H Lowe JK Chandran V. Integrative functional genomic analyses implicate specific molecular pathways and circuits in autism. Cell. (2013) 155:1008–21. 10.1016/j.cell.2013.10.031 24267887 PMC3934107

[44] Rubenstein J Merzenich MM. Model of autism: increased ratio of excitation/inhibition in key neural systems. Genes Brain Behav. (2003) 2:255–67. 10.1034/j.1601-183X.2003.00037.x 14606691 PMC6748642

[45] Nowakowski TJ Bhaduri A Pollen AA Alvarado B Mostajo-Radji MA Di Lullo E. Spatiotemporal gene expression trajectories reveal developmental hierarchies of the human cortex. Science. (2017) 358:1318–23. 10.1126/science.aap8809 29217575 PMC5991609

[46] Jacquemont S Coe BP Hersch M Duyzend MH Krumm N Bergmann S. A higher mutational burden in females supports a “female protective model” in neurodevelopmental disorders. Am J Hum Genet. (2014) 94:415–25. 10.1016/j.ajhg.2014.02.001 24581740 PMC3951938

[47] Ferri SL Abel T Brodkin ES. Sex differences in autism spectrum disorder: a review. Curr Psychiatry Rep. (2018) 20:9. 10.1007/s11920-018-0874-2 29504047 PMC6477922

[48] Olivetti PR Maheshwari A Noebels JL. Neonatal estradiol stimulation prevents epilepsy in Arx model of X-linked infantile spasms syndrome. Sci Trans Med. (2014) 6:220ra212. 10.1126/scitranslmed.3007231 24452264 PMC4034383

[49] Turner TN Wilfert AB Bakken TE Bernier RA Pepper MR Zhang Z. Sex-based analysis of *de novo* variants in neurodevelopmental disorders. Am J Hum Genet. (2019) 105:1274–85. 10.1016/j.ajhg.2019.11.003 31785789 PMC6904808

[50] Gockley J Willsey AJ Dong S Dougherty JD Constantino JN Sanders SJ. The female protective effect in autism spectrum disorder is not mediated by a single genetic locus. Mol Autism. (2015) 6:25. 10.1186/s13229-015-0014-3 25973162 PMC4429476

[51] Werling DM Parikshak NN Geschwind DH. Gene expression in human brain implicates sexually dimorphic pathways in autism spectrum disorders. Nat Commun. (2016) 7:10717. 10.1038/ncomms10717 26892004 PMC4762891

[52] Voineagu I Wang X Johnston P Lowe JK Tian Y Horvath S. Transcriptomic analysis of autistic brain reveals convergent molecular pathology. Nature. (2011) 474:380–4. 10.1038/nature10110 21614001 PMC3607626

[53] Gupta S Ellis SE Ashar FN Moes A Bader JS Zhan J. Transcriptome analysis reveals dysregulation of innate immune response genes and neuronal activity-dependent genes in autism. Nat Commun. (2014) 5:5748. 10.1038/ncomms6748 25494366 PMC4270294

[54] Jack A Sullivan CAW Aylward E Bookheimer SY Dapretto M Gaab N. A neurogenetic analysis of female autism. Brain. (2021) 144:1911–26. 10.1093/brain/awab064 33860292 PMC8320285

[55] Kaiser MD Hudac CM Shultz S Lee SM Cheung C Berken AM. Neural signatures of autism. Proc Natl Acad Sci USA. (2010) 107:21223–8. 10.1073/pnas.1010412107 21078973 PMC3000300

[56] Baron-Cohen S. The extreme male brain theory of autism. Trends Cogn Sci. (2002) 6:248–54. 10.1016/S1364-6613(02)01904-6 12039606

[57] Baron-Cohen S Lombardo MV Auyeung B Ashwin E Chakrabarti B Knickmeyer R. Why are autism spectrum conditions more prevalent in males? PLoS Biol. (2011) 9:e1001081. 10.1371/journal.pbio.1001081 21695109 PMC3114757

[58] Auyeung B Baron-Cohen S Ashwin E Knickmeyer R Taylor K Hackett G. Fetal testosterone and autistic traits. Br J Psychol. (2009) 100:1–22. 10.1348/000712608X311731 18547459

[59] Auyeung B Taylor K Hackett G Baron-Cohen S. Foetal testosterone and autistic traits in 18 to 24-month-old children. Mol Autism. (2010) 1:11. 10.1186/2040-2392-1-11 20678186 PMC2916006

[60] Baron-Cohen S Auyeung B Nørgaard-Pedersen B Hougaard DM Abdallah MW Melgaard L. Elevated fetal steroidogenic activity in autism. Mol Psychiatry. (2015) 20:369–76. 10.1038/mp.2014.48 24888361 PMC4184868

[61] Baron-Cohen S Tsompanidis A Auyeung B Nørgaard-Pedersen B Hougaard DM Abdallah M. Foetal oestrogens and autism. Mol Psychiatry. (2019) 25:2970–8. 10.1038/s41380-019-0454-9 31358906 PMC7577840

[62] Bilder DA Esplin MS Coon H Burghardt P Clark EAS Fraser A. Early second trimester maternal serum steroid-related biomarkers associated with autism spectrum disorder. J Autism Dev Disord. (2019) 49:4572–83. 10.1007/s10803-019-04162-2 31410696 PMC6814559

[63] Sharpe MA Gist TL Baskin DS. Alterations in sensitivity to estrogen, dihydrotestosterone, and xenogens in B-lymphocytes from children with autism spectrum disorder and their unaffected twins/siblings. J Toxicol. (2013) 2013:159810. 10.1155/2013/159810 24363669 PMC3836453

[64] Altun H Kurutaş EB Sahin N Sinir H Findikli E. Decreased levels of G protein-coupled estrogen receptor in children with autism spectrum disorders. Psychiatry Res. (2017) 257:67–71. 10.1016/j.psychres.2017.06.008 28734238

[65] Sarachana T Xu M Wu R-C Hu VW. Sex hormones in autism: androgens and estrogens differentially and reciprocally regulate RORA, a novel candidate gene for autism. PLoS ONE. (2011) 6:e17116. 10.1371/journal.pone.0017116 21359227 PMC3040206

[66] Crider A Thakkar R Ahmed AO Pillai A. Dysregulation of estrogen receptor beta (ERβ), aromatase (CYP19A1), and ER co-activators in the middle frontal gyrus of autism spectrum disorder subjects. Mol Autism. (2014) 5:46. 10.1186/2040-2392-5-46 25221668 PMC4161836

[67] Knickmeyer R Baron-Cohen S Raggatt P Taylor K Hackett G. Fetal testosterone and empathy. Horm Behav. (2006) 49:282–92. 10.1016/j.yhbeh.2005.08.010 16226265

[68] Kosidou K Dalman C Widman L Arver S Lee BK Magnusson C. Maternal polycystic ovary syndrome and the risk of autism spectrum disorders in the offspring: a population-based nationwide study in Sweden. Mol Psychiatry. (2016) 21:1441–8. 10.1038/mp.2015.183 26643539 PMC5030459

[69] Tartaglia NR Wilson R Miller JS Rafalko J Cordeiro L Davis S. Autism spectrum disorder in males with sex chromosome aneuploidy: XXY/klinefelter syndrome, XYY, and XXYY. J Dev Behav Pediatr. (2017) 38:197–207. 10.1097/DBP.0000000000000429 28333849 PMC5423728

[70] Cederlöf M Ohlsson Gotby A Larsson H Serlachius E Boman M Långström N. Klinefelter syndrome and risk of psychosis, autism and ADHD. J Psychiatr Res. (2014) 48:128–30. 10.1016/j.jpsychires.2013.10.001 24139812

[71] Wolstencroft J Mandy W Skuse D. 040 Autism spectrum disorders in girls and women with turner syndrome. Arch Dis Child. (2018) 103:A16. 10.1136/goshabs.40

[72] Erli F Palmos AB Raval P Mukherjee J Sellers KJ Gatford NJF. Estradiol reverses excitatory synapse loss in a cellular model of neuropsychiatric disorders. Transl Psychiatry. (2020) 10:16. 10.1038/s41398-020-0682-4 32066698 PMC7026123

[73] Strauss KA Puffenberger EG Huentelman MJ Gottlieb S Dobrin SE Parod JM. Recessive symptomatic focal epilepsy and mutant contactin-associated protein-like 2. N Engl J Med. (2006) 354:1370–7. 10.1056/NEJMoa052773 16571880

[74] Ijaz S Hoffman EJ. Zebrafish: a translational model system for studying neuropsychiatric disorders. J Am Acad Child Adolesc Psychiatry. (2016) 55:746–8. 10.1016/j.jaac.2016.06.008 27566113 PMC5521170

[75] Sakai C Ijaz S Hoffman EJ. Zebrafish models of neurodevelopmental disorders: past, present, and future. Front Mol Neurosci. (2018) 11:294. 10.3389/fnmol.2018.00294 30210288 PMC6123572

[76] Peñagarikano O Abrahams BS Herman EI Winden KD Gdalyahu A Dong H. Absence of CNTNAP2 leads to epilepsy, neuronal migration abnormalities, and core autism-related deficits. Cell. (2011) 147:235–46. 10.1016/j.cell.2011.08.040 21962519 PMC3390029

[77] Rihel J Prober DA Arvanites A Lam K Zimmerman S Jang S. Zebrafish behavioral profiling links drugs to biological targets and rest/wake regulation. Science. (2010) 327:348–51. 10.1126/science.1183090 20075256 PMC2830481

[78] Prober DA Rihel J Onah AA Sung R-J Schier AF. Hypocretin/orexin overexpression induces an insomnia-like phenotype in zebrafish. J Neurosci. (2006) 26:13400. 10.1523/JNEUROSCI.4332-06.2006 17182791 PMC6675014

[79] Azuma K Inoue S. Genomic and non-genomic actions of estrogen: recent developments. Biomol Concepts. (2012) 3:365–70. 10.1515/bmc-2012-0002 25436542

[80] Raz L Khan MM Mahesh VB Vadlamudi RK Brann DW. Rapid estrogen signaling in the brain. Neurosignals. (2008) 16:140–53. 10.1159/000111559 18253054

[81] Eyster KM. The estrogen receptors: an overview from different perspectives. Methods Mol Biol. (2016) 1366:1–10. 10.1007/978-1-4939-3127-9_1 26585122

[82] MizukaMi Y. *In vivo* functions of GPR30/GPER-1, a membrane receptor for estrogen: from discovery to functions *in vivo*. Endocr J. (2009) 57:101–7. 10.1507/endocrj.K09E-332 19996532

[83] Revankar CM Cimino DF Sklar LA Arterburn JB Prossnitz ER. A transmembrane intracellular estrogen receptor mediates rapid cell signaling. Science. (2005) 307:1625–30. 10.1126/science.1106943 15705806

[84] Frick KM. Molecular mechanisms underlying the memory-enhancing effects of estradiol. Horm Behav. (2015) 74:4–18. 10.1016/j.yhbeh.2015.05.001 25960081 PMC4573242

[85] Luo J Liu D. Does GPER really function as a G protein-coupled estrogen receptor *in vivo*? Front Endocrinol. (2020) 11:148. 10.3389/fendo.2020.00148 32296387 PMC7137379

[86] Dominguez R Liu R Baudry M. 17-β-Estradiol-mediated activation of extracellular-signal regulated kinase, phosphatidylinositol 3-kinase/protein kinase B-Akt and N-methyl-d-aspartate receptor phosphorylation in cortical synaptoneurosomes. J Neurochem. (2007) 101:232–40. 10.1111/j.1471-4159.2006.04360.x 17250656 PMC3182115

[87] Srivastava DP Penzes P. Rapid estradiol modulation of neuronal connectivity and its implications for disease. Front Endocrinol. (2011) 2:77. 10.3389/fendo.2011.00077 22654827 PMC3356153

[88] Srivastava DP Woolfrey KM Jones KA Shum CY Lash LL Swanson GT. Rapid enhancement of two-step wiring plasticity by estrogen and NMDA receptor activity. Proc Nat Acad Sci. (2008) 105:14650–5. 10.1073/pnas.0801581105 18801922 PMC2567160

[89] Mannella P Brinton RD. Estrogen receptor protein interaction with phosphatidylinositol 3-kinase leads to activation of phosphorylated Akt and extracellular signal-regulated kinase 1/2 in the same population of cortical neurons: a unified mechanism of estrogen action. J Neurosci. (2006) 26:9439–47. 10.1523/JNEUROSCI.1443-06.2006 16971528 PMC6674594

[90] Lu Y Sareddy GR Wang J Wang R Li Y Dong Y. Neuron-derived estrogen regulates synaptic plasticity and memory. J Neurosci. (2019) 39:2792–809. 10.1523/JNEUROSCI.1970-18.2019 30728170 PMC6462452

[91] Berry-Kravis E Hicar M Ciurlionis R. Reduced cyclic AMP production in fragile X syndrome: cytogenetic and molecular correlations. Pediatr Res. (1995) 38:638–43. 10.1203/00006450-199511000-00002 8552427

[92] Berry-Kravis E Huttenlocher PR. Cyclic AMP metabolism in fragile X syndrome. Ann Neurol. (1992) 31:22–6. 10.1002/ana.410310105 1371909

[93] Berry-Kravis EM Harnett MD Reines SA Reese MA Ethridge LE Outterson AH. Inhibition of phosphodiesterase-4D in adults with fragile X syndrome: a randomized, placebo-controlled, phase 2 clinical trial. Nat Med. (2021) 27:862–70. 10.1038/s41591-021-01321-w 33927413

[94] Grissom NM McKee SE Schoch H Bowman N Havekes R O'Brien WT. Male-specific deficits in natural reward learning in a mouse model of neurodevelopmental disorders. Mol Psychiatry. (2018) 23:544–55. 10.1038/mp.2017.184 29038598 PMC5822461

[95] Nuñez JL Bambrick LL Krueger BK McCarthy MM. Prolongation and enhancement of gamma-aminobutyric acid receptor mediated excitation by chronic treatment with estradiol in developing rat hippocampal neurons. Eur J Neurosci. (2005) 21:3251–61. 10.1111/j.1460-9568.2005.04175.x 16026463

[96] Perrot-Sinal TS Sinal CJ Reader JC Speert DB McCarthy MM. Sex differences in the chloride cotransporters, NKCC1 and KCC2, in the developing hypothalamus. J Neuroendocrinol. (2007) 19:302–8. 10.1111/j.1365-2826.2007.01530.x 17355320

[97] Amantea D Russo R Bagetta G Corasaniti MT. From clinical evidence to molecular mechanisms underlying neuroprotection afforded by estrogens. Pharmacol Res. (2005) 52:119–32. 10.1016/j.phrs.2005.03.002 15967377

[98] Wise PM. Estrogens and neuroprotection. Trends Endocrinol Metab. (2002) 13:229–30. 10.1016/S1043-2760(02)00611-2 12128278

[99] Singer CA Figueroa-Masot XA Batchelor RH Dorsa DM. The mitogen-activated protein kinase pathway mediates estrogen neuroprotection after glutamate toxicity in primary cortical neurons. J Neurosci. (1999) 19:2455–63. 10.1523/JNEUROSCI.19-07-02455.1999 10087060 PMC6786088

[100] Honda K Sawada H Kihara T Urushitani M Nakamizo T Akaike A. Phosphatidylinositol 3-kinase mediates neuroprotection by estrogen in cultured cortical neurons. J Neurosci Res. (2000) 60:321–7. 10.1002/(SICI)1097-4547(20000501)60:3<321::AID-JNR6>3.0.CO 10797534

[101] Honda K Shimohama S Sawada H Kihara T Nakamizo T Shibasaki H. Nongenomic antiapoptotic signal transduction by estrogen in cultured cortical neurons. J Neurosci Res. (2001) 64:466–75. 10.1002/jnr.1098 11391701

[102] Biran J Levkowitz G. Zebrafish reel in phenotypic suppressors of autism. Neuron. (2016) 89:673–5. 10.1016/j.neuron.2016.02.005 26889805

[103] Choleris E Gustafsson J-Å Korach KS Muglia LJ Pfaff DW Ogawa S. An estrogen-dependent four-gene micronet regulating social recognition: a study with oxytocin and estrogen receptor-α and -β knockout mice. Proc Nat Acad Sci. (2003) 100:6192. 10.1073/pnas.0631699100 12730370 PMC156348

[104] Beyer C Karolczak M. Estrogenic stimulation of neurite growth in midbrain dopaminergic neurons depends on cAMP/protein kinase A signalling. J Neurosci Res. (2000) 59:107–16. 10.1002/(SICI)1097-4547(20000101)59:1<107::AID-JNR13>3.0.CO;2-W 10658191

[105] Hill RA Pompolo S Jones MEE Simpson ER Boon WC. Estrogen deficiency leads to apoptosis in dopaminergic neurons in the medial preoptic area and arcuate nucleus of male mice. Mol Cell Neurosci. (2004) 27:466–76. 10.1016/j.mcn.2004.04.012 15555924

[106] Waters EM Simerly RB. Estrogen induces caspase-dependent cell death during hypothalamic development. J Neurosci. (2009) 29:9714–8. 10.1523/JNEUROSCI.0135-09.2009 19657024 PMC6428191

[107] Chaban VV Lakhter AJ Micevych P. A membrane estrogen receptor mediates intracellular calcium release in astrocytes. Endocrinology. (2004) 145:3788–95. 10.1210/en.2004-0149 15131017

[108] Dhandapani KM Wade FM Mahesh VB Brann DW. Astrocyte-derived transforming growth factor-β mediates the neuroprotective effects of 17β-estradiol: involvement of nonclassical genomic signaling pathways. Endocrinology. (2005) 146:2749–59. 10.1210/en.2005-0014 15746252

[109] Lenz KM Nugent BM Haliyur R McCarthy MM. Microglia are essential to masculinization of brain and behavior. J Neurosci. (2013) 33:2761–72. 10.1523/JNEUROSCI.1268-12.2013 23407936 PMC3727162

[110] Srivastava DP Waters EM Mermelstein PG Kramár EA Shors TJ Liu F. Rapid estrogen signaling in the brain: implications for the fine-tuning of neuronal circuitry. J Neurosci. (2011) 31:16056–63. 10.1523/JNEUROSCI.4097-11.2011 22072656 PMC3245715

[111] Gresack JE Frick KM. Post-training estrogen enhances spatial and object memory consolidation in female mice. Pharmacol Biochem Behav. (2006) 84:112–9. 10.1016/j.pbb.2006.04.013 16759685

[112] Boulware MI Heisler JD Frick KM. The memory-enhancing effects of hippocampal estrogen receptor activation involve metabotropic glutamate receptor signaling. J Neurosci. (2013) 33:15184–94. 10.1523/JNEUROSCI.1716-13.2013 24048848 PMC6618419

[113] Boulware MI Weick JP Becklund BR Kuo SP Groth RD Mermelstein PG. Estradiol activates group I and II metabotropic glutamate receptor signaling, leading to opposing influences on cAMP response element-binding protein. J Neurosci. (2005) 25:5066. 10.1523/JNEUROSCI.1427-05.2005 15901789 PMC6724851

[114] Fan L Zhao Z Orr PT Chambers CH Lewis MC Frick KM. Estradiol-induced object memory consolidation in middle-aged female mice requires dorsal hippocampal extracellular signal-regulated kinase and phosphatidylinositol 3-kinase activation. J Neurosci. (2010) 30:4390. 10.1523/JNEUROSCI.4333-09.2010 20335475 PMC2852263

[115] Fernandez SM Lewis MC Pechenino AS Harburger LL Orr PT Gresack JE. Estradiol-induced enhancement of object memory consolidation involves hippocampal extracellular signal-regulated kinase activation and membrane-bound estrogen receptors. J Neurosci. (2008) 28:8660. 10.1523/JNEUROSCI.1968-08.2008 18753366 PMC2693006

[116] Fortress AM Fan L Orr PT Zhao Z Frick KM. Estradiol-induced object recognition memory consolidation is dependent on activation of mTOR signaling in the dorsal hippocampus. Learn Mem. (2013) 20:147–55. 10.1101/lm.026732.112 23422279 PMC3578274

[117] Zhao Z Fan L Frick KM. Epigenetic alterations regulate estradiol-induced enhancement of memory consolidation. Proc Nat Acad Sci. (2010) 107:5605. 10.1073/pnas.0910578107 20212170 PMC2851775

[118] Zhao Z Fan L Fortress AM Boulware MI Frick KM. Hippocampal histone acetylation regulates object recognition and the estradiol-induced enhancement of object recognition. J Neurosci. (2012) 32:2344–51. 10.1523/JNEUROSCI.5819-11.2012 22396409 PMC3401048

